# Exploring associations of adverse childhood experiences with patterns of 11 health risk behaviors in Chinese adolescents: focus on gender differences

**DOI:** 10.1186/s13034-023-00575-1

**Published:** 2023-02-20

**Authors:** Huiqiong Xu, Xinyu Zhang, Jiaojiao Wang, Yang Xie, Yi Zhang, Shaojun Xu, Yuhui Wan, Fangbiao Tao

**Affiliations:** 1 Department of Maternal, Child and Adolescent Health, School of Public Health, Anhui Medical University, No 81 Meishan Road, Hefei, 230032 Anhui China; 2 Key Laboratory of Population Health Across Life Cycle (Anhui Medical University), Ministry of Education of the People’s Republic of China, No 81 Meishan Road, Hefei, 230032 Anhui China; 3 NHC Key Laboratory of Study on Abnormal Gametes and Reproductive Tract, No 81 Meishan Road, Hefei, 230032 Anhui China; 4 Anhui Provincial Key Laboratory of Population Health and Aristogenics, 81 Meishan Road, Hefei, 230032 Anhui China

**Keywords:** Adverse childhood experiences, Health risk behaviors, Adolescent, Gender, Latent class analysis

## Abstract

**Purpose:**

Adolescents exposed to adverse childhood experiences (ACEs) are at increased risk for health-compromising behaviors. However, few studies have investigated how ACEs correlate with patterns of health risk behaviors (HRBs) during adolescence, a crucial developmental period. The aim was to extend the current knowledge about the relationship between ACEs and HRB patterns among adolescents, and to explore gender differences.

**Methods:**

A multi-centered population-based survey was conducted in 24 middle schools in three provinces across China between 2020 and 2021. A total of 16,853 adolescents effectively completed anonymous questionnaires covering exposure to eight ACE categories and 11 HRBs. Clusters were identified using latent class analysis. Logistic regression models were utilized to test the association between them.

**Results:**

There were four classes of HRB patterns: “Low all” (58.35%), “Unhealthy lifestyle” (18.23%), “Self-harm” (18.42%), and “High all” (5.0%). There were significant differences between HRB patterns in terms of the different numbers and types of ACEs in three logistic regression models. Specifically, compared to “Low all,” different types of ACEs were positively associated with the three other HRB patterns, and there were significant trends toward increase in the three latent classes of HRBs with higher ACEs. In general, females with ACEs had a higher risk of “High all” except sexual abuse than males.

**Conclusion:**

Our study comprehensively considers the association between ACEs and aggregation categories of HRBs. The results support efforts to improve clinical healthcare, and future work may explore protective factors based on individual, family, and peer education to mitigate the negative trajectory of ACEs.

**Supplementary Information:**

The online version contains supplementary material available at 10.1186/s13034-023-00575-1.

## Introduction

Adolescence is a period of opportunity and challenge. Adolescents have opportunities to learn new things in their lives, but compared with adults, they are more likely to engage in risk-taking behaviors that could expose them to negative consequences [1]. These behaviors are commonly known as health risk behaviors (HRBs). A school-based study reported that the rates of smoking, drinking, high screen time, non-suicidal self-injury (NSSI) and suicidal behaviors was 2.8%, 16.8%, 16.3%, 32.1% and 15.0% among Chinese adolescents, respectively [2]. Also, studies have shown that the behaviors and lifestyles formed in adolescence will maintain a trajectory and continue into adulthood to affect lifelong health [3]. Moreover, adolescent HRBs often do not exist alone; multiple behaviors tend to co-exist, such as smoking and alcohol, and physical inactivity, poor nutrition, and other behaviors of aggregation or coexistence, which has gradually attracted attention [4, 5]. This observation was also supported by the common liability model [6] and Jessor’s risk behavior theory [7]. The former noted that young adults who report heavy substance use tend toward polysubstance use [6, 8], which can be attributed to the influence of a common liability, including genetic vulnerability, family liability, as well as individual vulnerability such as adverse childhood experiences (ACEs) [9]. Jessor [7] emphasized that HRBs tend to co-occur in the youth perhaps because they share a common motivation of thrill seeking. Therefore, it is important to investigate the clustering of HRBs because individuals who engage in multiple HRBs are at greater risk of chronic physical diseases and mental health problems [10, 11].

Recent, person-center approaches have been used to describe HRB patterns over time [12, 13], and to support the common liability model and Jessor’s risk behavior theory. A longitudinal study among 853 Australia young adults assessed six risk behaviors (binge drinking and smoking in past 6 months, moderate-to-vigorous physical activity/week, sitting time/day, fruit and vegetable intake/day, and sleep duration/night); three classes emerged in the latent class analysis (LCA): “moderate risk,” “inactive, non-smokers,” and “smokers and binge drinkers,” respectively [10]. In addition, an investigation of the clustering of five HRBs (including smoking/alcohol use/screen time/unhealthy losing weight/problematic mobile phone use) among Chinese school adolescents identified four latent classes: low-risk, moderate-risk class 1, moderate-risk class 2, and high-risk [14]. To date, most studies investigating risk clusters have not included fast food, takeaways, and multiple beverage behaviors in their analyses [2, 15]. Thus, this study was further expanded to examine the clustering of 11 HRBs—smoking, alcohol use, takeaways, fast foods, carbonated drinks, sugared drinks, screen time, NSSI, suicidal ideation, suicide plan, and attempted suicide. This study makes an important contribution to the literature by examining traditional chronic disease risk behaviors (smoking, alcohol use) in combination with emerging risk behaviors (such as screen time and fast food delivery).

ACEs refer to a wide range of negative childhood experiences, including abuse (emotional, physical, or sexual), neglect (emotional or physical), violence between community or peers, and household dysfunction such as parental death or separation, family substance use, and witnessed domestic violence [16]. An alarming number of adolescents experience some form of ACEs. Recently, in a representative national survey of over 15,278 school adolescents aged between 10 and 20 years in China, 89.4% reported one or more categories of ACEs, with nearly half (46.3%) reporting three or more types of ACEs [17]. Specifically, nearly 45.7% reported emotional abuse, 20.3% reported physical abuse, 13.3% reported sexual abuse, 64.2% reported emotional neglect, 58.5% reported physical neglect, and 42.5% reported household dysfunction. Ample evidence has supported significant and enduring associations between ACEs and unhealthy behaviors and lifestyles [18]. Some studies suggested that ACEs act as triggering events to depression and suicide attempts in adulthood [19], creating a lifelong vulnerability to stress. Moreover, ACEs may sensitize individuals to negative health effects later in life; previous toxic stress theories [20, 21] and cumulative risk models [22] suggest that stressful experiences, such as early childhood adversity, can act as catalysts for behavioral and physiological changes. Toxic stress theory stated that when individuals experience intense, frequent, or long-term stress, such as physical or emotional abuse, chronic neglect, caregiver substance abuse or mental illness, exposure to violence, family financial hardship, insufficient adult support, and so on, this chronically activated stress response system disrupted the development of brain structures and other organ systems and increased the risk of stress-related disorders and cognitive impairment [20, 21]. The cumulative risk model can be used to understand how traumatic experiences affect long-term outcomes, such as substance use, and it was assumed that various risks, ranging from traumatic events and victimization to poor interpersonal histories, tend to occur simultaneously and can accumulate, leading to a variety of adverse or negative outcomes [8, 22].

Currently, gender differences in adolescent HRBs are relatively consistent, with males generally reporting higher levels than females. Males reported higher overall levels of risky behavior compared to females from age 12 to 18 [3]. Theoretical explanations tend to focus on gender as a social status imposed on adolescents through the different socialization of males and females. In traditional Chinese culture, parents have different expectations and requirements from their sons and daughters, boys bear the responsibility of continuing the family line, and have higher “earning power” than girls in general [23]. Socialization of gender differentiation may enable boys to gain more autonomy and freedom to spend time outside, thus giving boys more opportunities to engage in risky behavior than girls. In addition, studies suggest that the gender differences between ACEs and risk behaviors are uncertain in adolescents [24, 25]. Therefore, different socio-cultural contexts may lead to gender differences in the association between ACEs and HRBs patterns.

In the current study, our first goal was to analyze the patterns of 11 HRBs based on a large sample in Chinese school adolescents. Furthermore, in line with prior work as well as toxic stress theory and cumulative risk models, we hypothesized that adolescents who reported ACEs were more likely to be classified into the more severe risk category than those who did not report ACEs. Finally, we also allow gender differences to be an exploratory aim, as few studies have investigated gender differences in the association of ACEs and HRB patterns, and thus we also hypothesized that female who reported ACEs were more likely to be classified into the more severe risk category than that of male.

## Methods

### Sample and procedures

The study population was recruited from the National Adolescent Health Surveillance Study, involving adolescents located in three cities of China between October 2020 and June 2021. First, both urban and rural regions from the northern (Beijing City), central (Zhengzhou, Henan Province), and southern (Yangjiang, Guangdong Province) parts of China were included to balance the level of economic development, geographical location, and demographic distribution. Second, two rural junior and senior schools and two urban junior and senior schools were randomly chosen to recruit participants in each target area, and 24 middle schools were included in all. Third, at least 200 students were selected from each grade, about 600 students were surveyed in each school, with a sample size of 4800 per region. Then, a total of 17,800 school adolescents (aged 11–19 years) from grades 7–12 were recruited and asked to complete an anonymous questionnaire. After obtaining the approval of the local school, we entered the classroom for investigation. Meanwhile, the teacher was responsible for maintaining order and not interfering with the answers. The investigators introduced the purpose of survey was to understand the mental and behavioral health conditions of Chinese adolescents, and pointed out aspects in the questionnaire that needed attention, as well as emphasized the principles of anonymity, confidentiality, and voluntary participation. About 30 min later, the completed questionnaires were collected and checked by investigator.

**Fig. 1:**
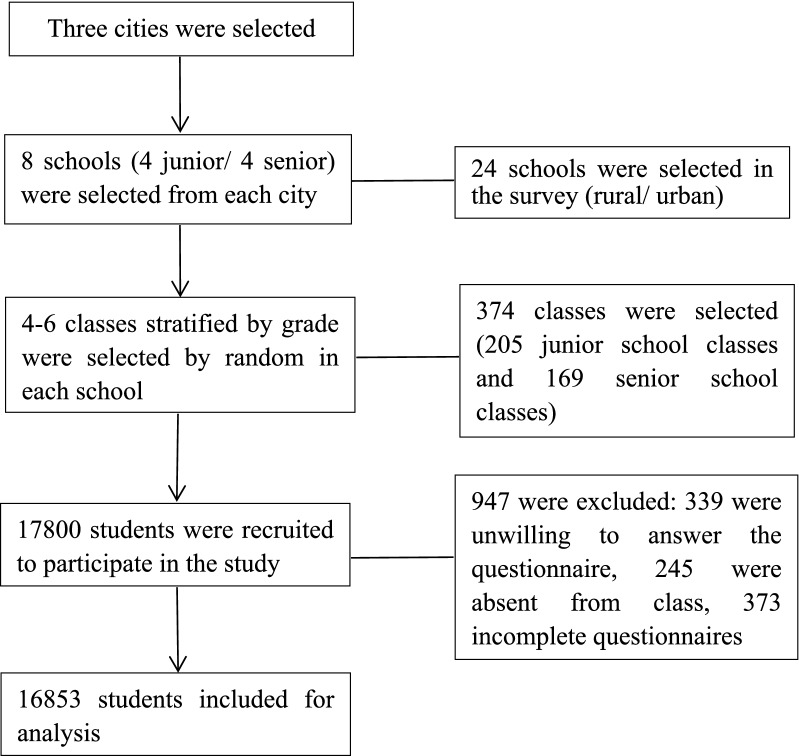
Flow chart presenting the selection of study participants

After screening, 947 invalid questionnaires were removed, because 1.9% (339) of the students or their parents/guardians were unwilling to participate in the study/investigation, 1.3% (245) students were absent on the day of the survey, and 2.1% (373) has incomplete questionnaires with a high level of missing data (> 15%) or apparent logic errors or inconsistent answers. Ultimately, 16,853 valid samples were obtained, at an efficiency rate of 94.7% (Flowchart shown in Fig. 1). The design and data collection procedure were approved by the Ethics Committee of Anhui Medical University (220200965). Informed consent was obtained from the participants or their parents/guardians, and they could opt out of the study at any time.

### Data collection

#### Demographic and control variables

The demographic characteristics were measured, including gender, grade, residency, only child or not, parents’ education level, family economic level, number of friends, and self-evaluation of academic performance.

As psychological symptoms are very important factors related to ACEs and HRBs, the Multi-dimensional Sub-health Questionnaire of Adolescents (MSQA) [26, 27] was used to evaluate psychological symptoms, including emotional problems, conduct problems, and social adaptation problems. The MSQA has 39 short questions (e.g., Do you always blame yourself?), each question had 6 options (6 = no or lasts less than 1 week, 5 = lasts more than 1 week, 4 = lasts 2 weeks or more, 3 = lasts for more than 1 month, 2 = lasts for more than 2 months, 1 = lasts for more than 3 months) were assigned 0–5 points, respectively. Items were summed to create a composite score, with higher scores indicating more obvious psychological symptoms. The good reliability and validity of the scale has been verified in previous studies [2, 28]. The Cronbach’s α coefficient was 0.938 in the present study.

#### Adverse childhood experiences

ACEs were defined across three domains: childhood maltreatment [29], violence outside the home [29] and household dysfunction [22]. Overall, these domains represent eight classes of ACEs. The scale has been translated into Chinese and applied in a previous study [30].

Childhood maltreatment included emotional and physical neglect and emotional, physical, and sexual abuse. Violence outside the home included community violence and peer bullying. The response to each item ranged across “never” “occasionally,” “sometimes,” “often,” or “always.” Responses were defined as “no” if they answered “never”; otherwise, it was defined as “yes” for this item. Household dysfunction was assessed through the following experiences: witnessed parental violence, parent or guardian died, lived with alcoholic or gambler, family members with major depression, mental illness or suicide, parent or guardian served time in prison, or parents had separated or divorced. Each entry had “yes” and “no” options. The Cronbach’s α coefficient was 0.777 in the present study. Finally, the dichotomized ACEs items were added to create a continuous number of ACE scores of 0 to 8; then the total score was divided into four categories (0, 1–2, 3–4, and 5–8), with “0” as the reference group for the analysis.

#### Health risk behaviors

##### Smoking and alcohol use

Based on the American Youth Risk Behavior Surveillance System (YRBSS) evaluation of current smoking and alcohol use [29], we focused on “How many days did you smoke cigarettes in the past 30 days” and “How many days did you have at least one drink of alcohol in the past 30 days.” Answers were on a scale from 1 = none to 2 = 1–2 days, 3 = 3–5 days, 4 = 6–9 days, 5 = 10–19 days, 6 = 20–29 days, and 7 = every day. Thus, we derived dichotomous indicators, “1 = none” as no and other options as yes, which was considered as having smoking or alcohol use behaviors.

##### Takeaways and fast foods

Takeaway consumption was assessed by asking “How many times have you eaten takeaways during the last week (e.g., through Ele.me, Meituan apps, etc.)” and fast food consumption was evaluated by asking “How many times have you eaten fast food in the last 7 days (e.g., fried chicken, barbecue, etc.)” [28, 31]. For these two items, response options were 1 = none, 2 = 1–2 times, 3 = 3–4 times, and 4 = 5 or more times. This was dichotomized by terming ≤ 2 times as no and ≥ 3 times as yes.

##### Carbonated drinks and sugared drinks

The use of carbonated drinks [29] and sugared drinks [28, 31] was evaluated with the question “During the past 7 days, how many times did you usually drink a can, bottle, or glass of soda or pop (e.g., Coke, Pepsi, or Sprite, etc.)?” The question was also employed for sugared drinks (e.g., fruit and vegetable juice drinks, coconut milk, or Red bull, etc.). Seven response options ranged from 0 to 7 times, defined as yes for 3 times or more.

##### Screen time

The participants were asked “On school days, how much time did you spend watching videos or playing games, or doing things unrelated to study on computer every day on average?” According to the American Academy of Pediatrics standards [32], screen time > 2 h/day was defined as high screen time (defined as yes).

##### Non-suicidal self-injury

We used the Adolescent Non-suicidal Self-injury Assessment Questionnaire [33], consisting of 12 items. Participants were asked, “In the past year, have you committed any of the following intentional harm to yourself that was not intended to kill yourself but may cause bleeding, bruising, or pain (exclude actions taken to avoid fatigue). For example, did you intentionally pinch yourself? If no, select 1; if yes, select 2 and enter the number of times. Answer the other questions in the same way.” We then added the total frequency of the 12 items; frequency greater than or equal to 1 time was considered “yes.” Cronbach’s α in the present study was 0.919.

##### Suicidal ideation, suicide plan, and suicide attempt

Suicide behaviors were assessed from 3 items referring to the YRBSS [29]. The questions were “Did you ever seriously consider attempting suicide in the last year?” “Did you make a plan about how you would attempt suicide in the last year?” “How many times did you actually attempt suicide?” Each question had four selection categories: 1 = none; 2 = 1 time; 3 = 2–3 times; 4 = 4 times or more. An answer indicating one or more times was considered yes.

### Statistical analysis

All analyses were conducted in Mplus, version 7.4 (Muthén & Muthén, Los Angeles, CA, USA) and SPSS 23.0 (SPSS Inc, Chicago, IL, USA). Data analysis consisted of four parts.

First, the number of HRB classes were estimated by LCA, which emerged as modern and person-centered rather than variable-centered approaches [34]. It can help to explain population heterogeneity in the observed data by identifying potential subgroups of individuals, allowing for the examination of different HRBs while dealing with the diverse nature of the population [12, 14]. Model fit was evaluated with several indicators, such that better model fit was indicated by lower values of Akaike information criterion (AIC), Bayesian information criterion (BIC), sample-size adjusted Bayesian Information Criteria (aBIC), higher entropy (closer to “1”), and non-significant values for the Lo-Mendell-Rubin likelihood ratio test (LMR-LRT) and bootstrapped likelihood ratio tests (BLRT) [35]. Second, chi-squared tests were used to compare HRB patterns among different demographic variables. Third, we used multiple logistics regression to estimate associations between ACEs and HRB patterns. We initially unadjusted confounding factors (Model 1), then adjusted only for demographic confounders (gender, grade, residency, only child or not, parents’ education level, family economic level, number of friends, and self-evaluation of academic performance; Model 2), and then further adjusted for individual psychological symptoms (Model 3). Fully adjusted model effects were tested in different gender subgroups. Finally, the gender differences in the associations were examined via ratio of two odds ratios (RORs) [36].

## Results

### Prevalence of HRBs

In total, 3.6% and 10.8% of students reported smoking and alcohol use in the past month, respectively. During the last week, 12.9%, 19.0%, 21.3%, and 33.2% had a frequency of 3 times or more for consuming takeaways, fast foods, carbonated drinks, and sugared drinks, respectively. A total of 15.8% of participants reported spending at least 2 h screen time during the school day; 28.9% of the sample reported NSSI in the past year, with 31.3%, 16.3%, and 7.5% reporting suicidal ideation, suicide plan, and attempted suicide, respectively. Furthermore, 71.0% reported engaging in at least one form of HRBs, with 48.9% reporting more than one behavior (Table 1).

**Table 1:** The distribution of health risk behaviors

Number	*n*	*%*
0	4889	29.0
1	3721	22.1
2	3768	16.4
3	1991	11.8
4	1432	8.5
5–11	2052	12.2

### HRB pattern classification

As showed in Additional file 1: Table S1, models with 1 to 6 categories were tested in LCA. Four types of models were selected based on lower AIC, BIC, aBIC, and higher entropy (0.787), and the average posterior class membership probability score was acceptable between groups (0.803–0.919; Additional file 1: Table S2).

**Fig. 2:**
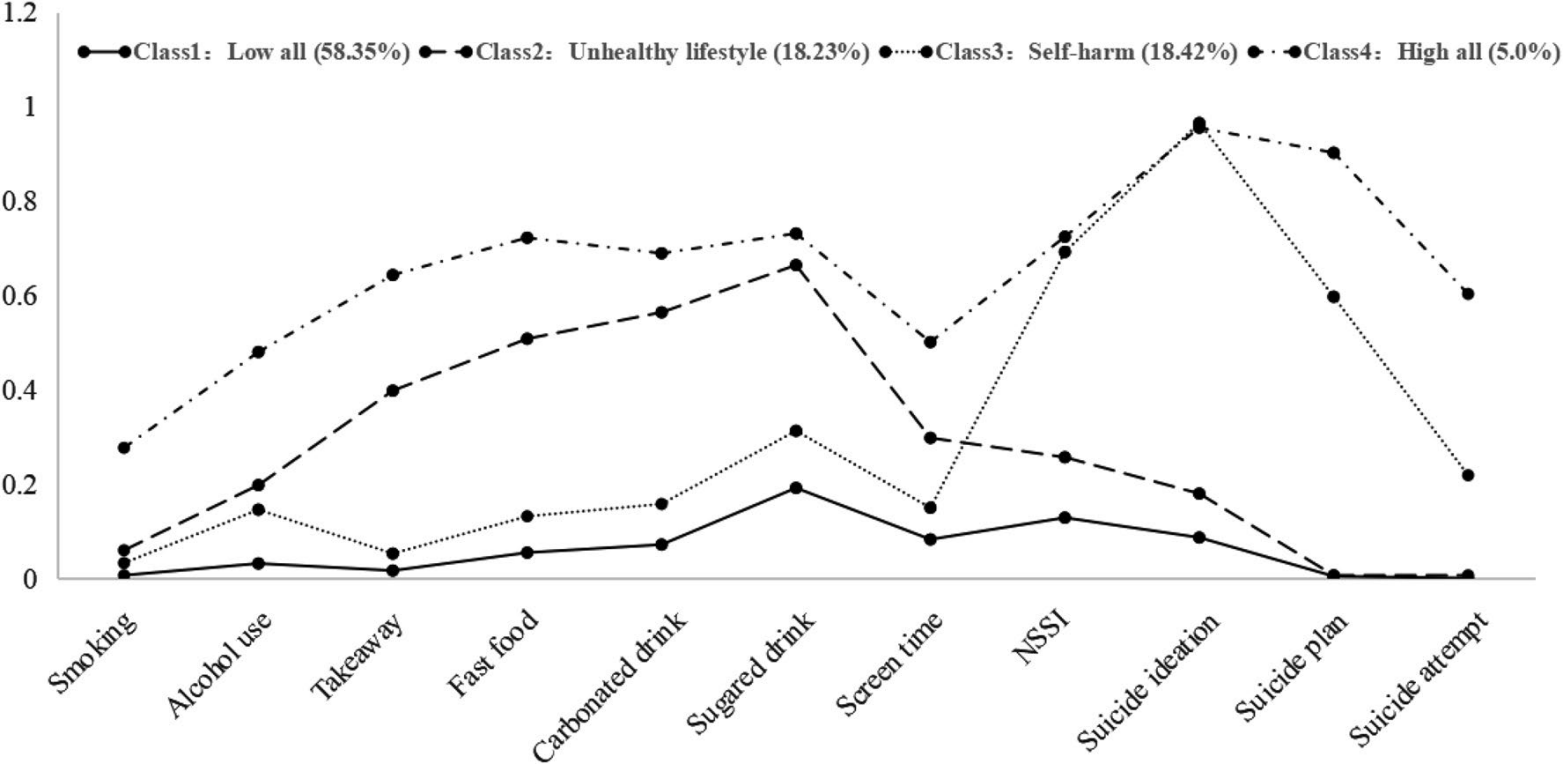
Plot of four latent classes of health risk behaviors

Figure 2 shows the four latent classes of HRBs. Class 1 was labeled as “Low all” (9833, 58.35%), which was characterized by a low probability of engaging in 11 HRBs. Class 2 was labeled as “unhealthy lifestyle” (3073, 18.23%), comprising participants who were more likely to engage in smoking, alcohol use, unhealthy eating behaviors, and high screen time, as well as slight risk of NSSI and suicidal behaviors. Class 3 was labeled as “Self-harm” (3104, 18.42%) with a higher risk of NSSI, suicidal ideation, suicide plan, and attempted suicide, as well as slight risk of smoking, alcohol use, unhealthy eating behaviors, and high screen time. In addition, Class 4 was labeled as “High all” (843, 5.0%), which was characterized by a high probability of exposure to 11 HRBs. In addition, following reviewer suggestions, we also analyzed the prevalence of specific HRBs within each class, see Additional file 1: Table S3 for details.

### Characteristics of participants by HRB patterns

The participants’ mean age was 14.7 ± 1.8 years; 49.8% were males (8390) and 50.2% were females (8463); 54.8% were junior school students (9235) and 45.2% were senior school students (7618), and see Table 2 for more information on the samples.

**Table 2:** Characteristics of the sample by health risk behavior patterns

Characteristics	N = 16,853	Class1	Class2	Class3	Class4	*χ*^*2*^	p-value
Gender						268.94	< 0.001
Male	8390 (49.8)	4856 (57.9)^a^	1869 (22.3)^b^	1240 (14.8)^c^	423 (5.0)^a^		
Female	8463 (50.2)	4977 (58.8)^a^	1204 (14.2)^b^	1862 (22.0)^c^	420 (5.0)^a^		
Grade						83.58	< 0.001
Junior school	9235 (54.8)	5187 (56.2)^a^	1685 (18.2)^a,b^	1793 (19.4)^b^	570 (6.2)^c^		
Senior school	7618 (45.2)	4646 (61.0)^a^	1388 (18.2)^a,b^	1311 (17.2)^b^	273 (3.6)^c^		
Residency						6.12	0.106
Rural	5805 (34.4)	3441 (59.3)^a^	1015 (17.5)^a^	1078 (18.6)^a^	271 (4.7)^a^		
Urban	11,048 (65.6)	6392 (57.9)^a^	2058 (18.6)^a^	2026 (18.3)^a^	572 (5.2)^a^		
Only child						51.15	< 0.001
Yes	5710 (33.9)	3261 (57.1)^a^	1164 (20.4)^b^	951 (16.7)^a^	334 (5.8)^b^		
No	11,143 (66.1)	6572 (59.0)^a^	1909 (17.1)^b^	2153 (19.3)^a^	509 (4.6)^b^		
Father’s education level						46.25	< 0.001
Primary or below	1830 (10.9)	1030 (56.3)^a^	288 (15.7)^a^	391 (21.4)^b^	121 (6.6)^b^		
Junior middle school	6211 (36.9)	3643 (58.7)^a^	1084 (17.5)^a^	1164 (18.7)^a^	320 (5.2)^a^		
Senior middle school	4902 (29.1)	2823 (57.6)^a^	979 (20.0)^b^	867 (17.7)^a^	233 (4.8)^a,b^		
College or above	3910 (23.2)	2337 (59.8)^a^	722 (18.5)^a^	682 (17.4)^a^	169 (4.3)^a^		
Mother’s education level						49.51	< 0.001
Primary or below	2423 (14.4)	1441 (59.5)^a^	354 (14.6)^b^	487 (20.1)^a^	141 (5.8)^a^		
Junior middle school	6151 (36.5)	3642 (59.2)^a^	1082 (17.6)^a^	1119 (18.2)^a^	308 (5.0)^a^		
Senior middle school	4634 (27.5)	2606 (56.2)^a^	914 (19.7)^b^	882 (19.0)^a,b^	232 (5.0)^b^		
College or above	3645 (21.6)	2144 (58.8)^a,b^	723 (19.8)^b^	616 (16.9)^a^	162 (4.4)^a^		
Family economic level						246.08	< 0.001
Poor	2119 (12.6)	1094 (51.6)^a^	287 (13.5)^b^	579 (27.3)^c^	159 (7.5)^c^		
Fair	11,677 (69.3)	7031 (60.2)^a^	2097 (18.0)^b^	2057 (17.6)^b^	492 (4.2)^c^		
Good	3057 (18.1)	1708 (55.9)^a^	689 (22.5)^b^	468 (15.3)^c^	192 (6.3)^b^		
Number of friends						538.55	< 0.001
0	553 (3.3)	228 (41.2)^a^	64 (11.6)^a^	164 (29.7)^b^	97 (17.5)^c^		
1–2	4096 (24.3)	2320 (56.6)^a^	573 (14.0)^b^	1002 (24.5)^c^	201 (4.9)^a^		
3–5	6949 (41.2)	4294 (61.8)^a^	1198 (17.2)^b^	1168 (16.8)^b^	289 (4.2)^b^		
≥ 6	5255 (31.2)	2991 (56.9)^a^	1238 (23.6)^b^	770 (14.7)^c^	256 (4.9)^a^		
Self-evaluation of academic performance						308.02	< 0.001
Poor	4087 (24.3)	1966 (48.1)^a^	800 (19.6)^b^	994 (24.3)^c^	327 (8.0)^d^		
Medium	10,316 (61.2)	6312 (61.2)^a^	1864 (18.1)^b^	1732 (16.8)^c^	408 (4.0)^d^		
Good	2450 (14.5)	1555 (63.5)^a^	409 (16.7)^b^	378 (15.4)^b^	108 (4.4)^a,b^		

Class1: Low all, Class2: Unhealthy lifestyle, Class3: Self-harm, Class4: High all

^a,b,c,d^There was no statistically significant difference between groups marked with the same letter

Also, Table 2 indicated the four HRB patterns among adolescents in different sociodemographic characteristics. Significant statistical differences in HRB patterns were observed among gender, grade, only child, parents’ education level, family economic level, number of friends and self-evaluation of academic performance in adolescents (all p < 0.001) except for residency area. The results of post hoc pairwise tests showed that there was statistically significant differences between the groups marked with different letters.

### ACEs and HRB patterns

#### Prevalence of ACEs

The most commonly reported ACE was emotional neglect (71.1%), followed by household dysfunction (38.3%), physical abuse (30.1%), emotional abuse (32.1%), peer bullying (28.8%), physical neglect (20.3%), community violence (15.6%), and sexual abuse (8.3%) among Chinese adolescents. Overall, 81.4% participants reported experiencing at least one form of ACEs, with 42.4% reporting three or more ACEs (Table 3).

**Table 3:** Association of adverse childhood experiences with health risk behavior patterns

ACEs	N = 16,853	Class2^*^		Class3^*^		Class4^*^	
		n (%)	*OR* (95% *CI*)^a^	n (%)	*OR* (95% *CI*)^a^	n (%)	*OR* (95% *CI*)^a^
Emotional neglect							
Yes	11,984 (71.1)	2134 (17.8)	1.17 (1.06–1.28)^#^	2661 (22.2)	2.46 (2.20–2.76)^**^	638 (5.3)	1.54 (1.30–1.83)^**^
No	4869 (28.9)	939 (19.3)	1.00	443 (9.1)	1.00	205 (4.2)	1.00
Physical neglect							
Yes	3426 (20.3)	616 (18.0)	1.30 (1.17–1.45)^**^	946 (27.6)	2.01 (1.82–2.21)^**^	321 (9.4)	2.85 (2.44–3.32)^**^
No	13,427 (79.7)	2457 (18.3)	1.00	2158 (16.1)	1.00	522 (3.9)	1.00
Emotional abuse							
Yes	5406 (32.1)	896 (16.6)	1.33 (1.21–1.46)^**^	1733 (32.1)	3.28 (3.00–3.58)^**^	479 (8.9)	3.59 (3.10–4.17)^**^
No	11,447 (67.9)	2177 (19.0)	1.00	1371 (12.0)	1.00	364 (3.2)	1.00
Physical abuse							
Yes	5079 (30.1)	833 (16.4)	1.20 (1.09–1.32)^**^	1530 (30.1)	2.62 (2.40–2.86)^**^	430 (8.5)	2.95 (2.55–3.42)^**^
No	11,774 (69.9)	2240 (19.0)	1.00	1574 (13.4)	1.00	413 (3.5)	1.00
Sexual abuse							
Yes	1396 (8.3)	213 (15.3)	1.25 (1.06–1.48)^#^	472 (33.8)	2.60 (2.27–2.98)^**^	176 (12.6)	3.88 (3.19–4.72)^**^
No	15,457 (91.7)	2860 (18.5)	1.00	2632 (17.0)	1.00	667 (4.3)	1.00
Community violence							
Yes	2631 (15.6)	455 (17.3)	1.41 (1.25–1.59)^**^	829 (31.5)	2.70 (2.43–3.01)^**^	303 (11.5)	4.42 (3.76–5.20)^**^
No	14,222 (84.4)	2618 (18.4)	1.00	2275 (16.0)	1.00	540 (3.8)	1.00
Peer bullying							
Yes	4852 (28.8)	771 (15.9)	1.14 (1.03–1.25)^**^	1544 (31.8)	2.73 (2.49–2.98)^**^	382 (7.9)	2.30 (1.98–2.67)^**^
No	12,001 (71.2)	2302 (19.2)	1.00	1560 (13.0)	1.00	461 (3.8)	1.00
Household dysfunction							
Yes	6460 (38.3)	1979 (19.0)	1.15 (1.05–1.25)^#^	1415 (13.6)	2.00 (1.83–2.18)^**^	364 (3.5)	2.34 (2.02–2.72)^**^
No	10,393 (61.7)	1094 (16.9)	1.00	1689 (26.1)	1.00	479 (7.4)	1.00
Numbers of ACEs							
5–8	3007 (17.8)	452 (15.0)	1.66 (1.43–1.93)^**^	1150 (38.2)	8.85 (7.41–10.56)^**^	361 (12.0)	6.20 (4.85–7.91)^**^
3–4	4138 (24.6)	749 (18.1)	1.29 (1.14–1.47)^**^	979 (23.7)	3.82 (3.22–4.54)^**^	195 (4.7)	1.72 (1.33–2.22)^**^
1–2	6576 (39.0)	1286 (19.6)	1.16 (1.04–1.30)^#^	786 (12.0)	1.84 (1.56–2.19)^**^	185 (2.8)	0.94 (0.73–1.21)
0	586 (18.6)	586 (18.7)	1.00	189 (6.0)	1.00	102 (3.3)	1.00

Class1: Low all, Class2: Unhealthy lifestyle, Class3: Self-harm, Class4: High all. ^*^ Class 1 was used as the reference category; ^a^ model 3: adjusted for grade, gender, residency, single child status, parents’ education level, family economic level, number of friends, self-evaluation of academic performance and psychological symptom; **p < 0.001; ^#^p < 0.05

#### Types of ACEs and HRB patterns, and gender differences

Adjusting for confounding factors before (Additional file 1: Fig. S1, Model 1) and after (Additional file 1: Fig. S2, Model 2; Table 3, Model 3), different types of ACEs were positively associated with different HRB patterns (p < 0.05 for each group vs. “Low all”). Compared with “Low all,” “Self-harm” was most associated with emotional neglect and peer bullying, followed by “High all” and “Unhealthy lifestyle.” The other types of ACEs had the greatest association with “High all,” followed by “Self-harm” and “Unhealthy lifestyle.”

**Fig. 3:**
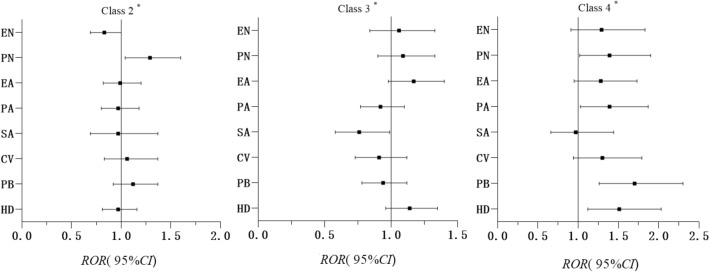
Association of ACEs with different latent classes of HRBs; ratio of two ORs (Model 3) in females vs males, ROR (95% CI). Adjusted for grade, gender, residency, single child status, parents’ education level, family economic level, number of friends, self-evaluation of academic performance, and psychological symptoms. Class 1: Low all, Class 2: Unhealthy lifestyle, Class 3: Self-harm, Class 4: High all. ^*^ Class 1 was used as the reference category; EN = emotional neglect, PN = physical neglect, EA = emotional abuse, PA = physical abuse, SA = sexual abuse, CV = community violence, PB = peer bullying, HD = household dysfunction

As shown in Additional file 1: Figs. S3 and S4, associations were observed among females and males (Model 3), which was similar to the total sample. Compared with “Low all,” when exposed to physical neglect, females were more likely to engage in “Unhealthy lifestyle” than males (ROR = 1.29, 95% CI = 1.04–1.60); the difference was statistically significant (p = 0.019) (Fig. 3). When exposed to physical neglect, physical abuse, peer bullying, and household dysfunction, females were more likely to engage in “High all” than males (ROR = 1.39, 95CI% = 1.02–1.90; ROR = 1.39, 95CI% = 1.03–1.87; ROR = 1.70, 95CI% = 1.26–2.30; ROR = 1.51, 95CI% = 1.12–2.03); the difference was statistically significant (p < 0.05).

#### Numbers of ACEs and HRB patterns, and gender difference

In Additional file 1: Table S4 (Model 1), compared with “Low all,” there were significant trends toward increase in other latent classes of HRBs with higher ACEs. Multiple adjusted odds ratios for other latent classes of HRBs were also significantly increased with higher ACEs (Additional file 1: Table S4, Model 2; Table 3, Model 3). Overall, in the three models, “Self-harm” had the highest OR, followed by “High all” and finally “Unhealthy lifestyle,” except that the association between 1–2 ACEs and “High all” was not statistically significant.

**Fig. 4:**
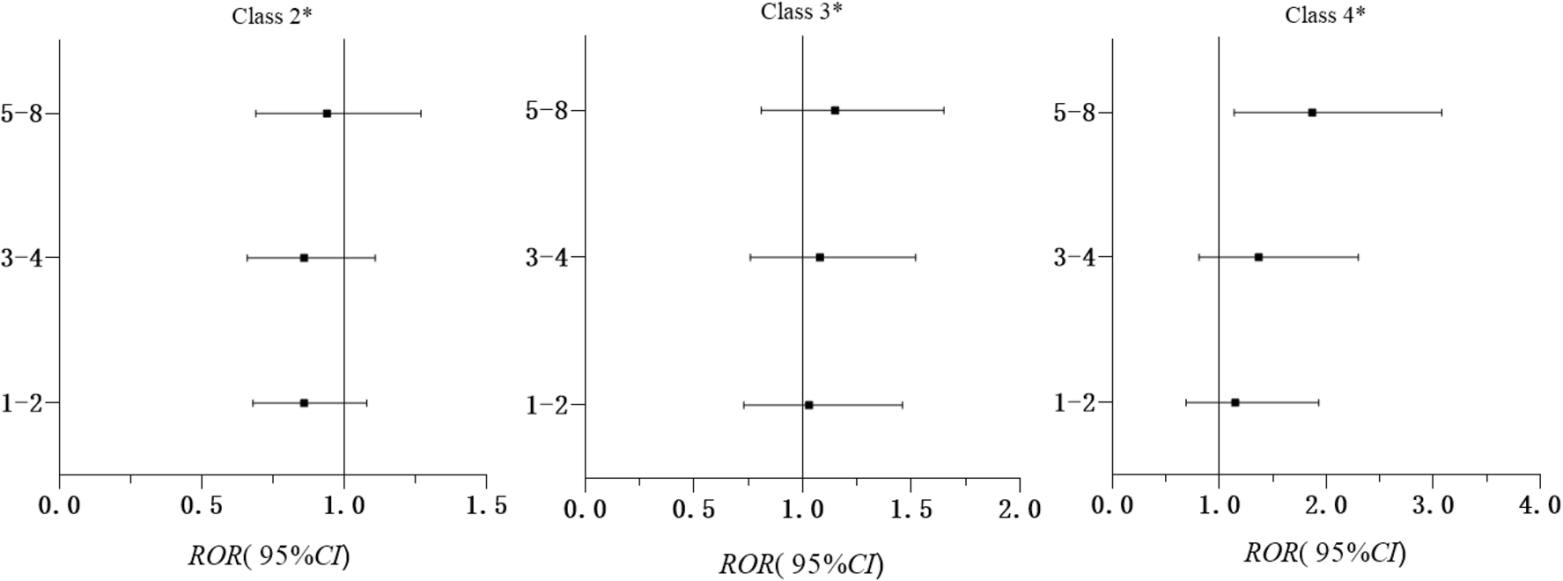
Relationship between numbers of ACEs and HRB patterns; ratio of two ORs (Model 3) in females vs males, ROR (95% CI). Adjusted for grade, gender, residency, single child status, parents’ education level, family economic level, number of friends, self-evaluation of academic performance and psychological symptoms. Class 1: Low all, Class 2: Unhealthy lifestyle, Class 3: Self-harm, Class 4: High all. ^*^ Class 1 was used as the reference category

In the female and male subgroups in Model 3, the cumulative exposure of ACEs according to the latent classes of HRBs are presented in Additional file 1: Figs. S5 and S6. As shown in Fig. 4, compared with “Low all,” females with 5–8 ACEs were more likely to engage in “High all” than males (ROR = 1.87, 95%CI = 1.14–3.08); the difference was statistically significant (p = 0.014).

## Discussion

Our study examined the effects of different types and numbers of ACEs on HRBs patterns in Chinese adolescents, as well as the gender differences. There were four latent classes of HRBs, namely “Low all” (Class 1), “Unhealthy lifestyle” (Class 2), “Self-harm” (Class 3), and “High all” (Class 4). In addition, our data revealed that participants with ACEs were more likely to have a severe HRB class, and similar relationships were found across genders. However, females were more likely to engage in “High all” when they had experienced physical neglect, physical abuse, peer bullying, and household dysfunction, as well as 5–8 ACEs.

### Four latent classes of HRBs

The first goal of this study was to find whether HRBs could be aggregated among our sample in meaningful risk profiles. Specifically, the LCA produced four patterns; the majority of participants belonged to “Low all” (58.35%); “Unhealthy lifestyle” (18.23%) and “Self-harm” (18.42%) had similar proportions; and a small group of participants was characterized as “High all” (5.0%). These clustering patterns are consistent with previous studies in this field. For instance, an investigation with 22,628 middle school students from six cities in China showed that four patterns of six behaviors could be identified: low-risk pattern (64.0%), substance use pattern (4.5%), injury pattern (28.8%), and high-risk pattern (2.7%) [2]. In addition, a systematic review found the strongest evidence for clustering of smoking and alcohol use [37]. It was worth noting that lifestyle-related behaviors, such as high screen time and unhealthy dietary behaviors, have been found to cluster with smoking and alcohol use in this study. So it might be useful to also examine in future study how strongly the individual behaviors are correlated with each other. Li et al. pointed out that screen time can be viewed as the use of electronic products, which is a kind of substance use [2]. In fact, it has been documented that certain foods may be addictive; those high in refined sugar, added salt, or added fat would be regarded as more addictive than foods without these ingredients [38]; therefore, the classification is also reasonable. Thus, this indirectly underscored that tighter controls on diet-related behavioral aspects may be as important as controlling substance use such as smoking and alcohol use in the future.

### Characteristics of HRB patterns

Interestingly, among males, the rates of “Unhealthy lifestyle” and “Self-harm” were 22.3% and 14.2%, respectively. In contrast, among females, the rates of “Unhealthy lifestyle” and “Self-harm” were 14.8% and 22.0%, respectively. The better the family economic level, the higher was the unhealthy lifestyle, and lower was self-harming behavior. Likewise, having more friends was associated with more unhealthy eating behaviors and less self-harming behaviors. Taking these results into account, we can see that gender, peer relationships, and family factors may have some influence on adolescent development. In addition, based on socio-ecological models [39] and Bronfenbrenner ecosystem theory [40], individual, family, peer relationships, and society are all related to adolescent health. Thus, it reminds the importance of adolescent peer relationships, and some actions could be taken to improve the training of interpersonal skills for adolescent, or focus on the psychological and behavioral problems of isolated students in schools. At the same time, we should pay more attention to the negative factors in the family, and enhance family bonds and resilience [41]. Finally, this suggests that gender-specific programs may be more in developing interventions or peer education, for example, it is more important for males to develop healthy lifestyle, while females should prevent self-injurious behaviors.

### The effects of ACEs on HRB patterns

In this study, 81.4% of adolescents were reported with ACEs exposure, which were slightly lower than the results of previous studies [17, 25], but higher than the results of Qu et al. in China [42]. To our knowledge, how ACEs were measured and evaluated also varied widely across Chinese ACE studies, the rate of exposure to at least one ACEs varied widely from 35.1% to 89.4% [17, 25, 42, 43]. One possible reason for the large difference in reporting rates was the diversity of the participant sample, but another, potentially more convincing reason, was the differences in inclusion and measurement of ACE across studies. These finding suggested that the overall prevalence of ACEs reported by Chinese adolescents was higher compared with international norms [44, 45]. For example, according to the prospective data from the New England Study of Suburban Youth (NESSY), which from relatively affluent Northeastern suburbs, 59.2% of participants reported at least one ACE and 14.2% reported three or more ACEs [44]. Croft J et al. used data from the Avon Longitudinal Study of Parents and Children, a large population-based birth cohort in the United Kingdom, 64.5% of the imputed sample reported exposure to trauma between 0 and 17 years of age [45]. However, Another study found that 84% reported at least one ACE between birth and 16 years [46]. It is possible that these differences stemmed from how ACEs were measured across studies. Specifically, the NESSY cohort only included three kinds of ACEs (e.g. parental criticism, parental divorce/separation and parental neglect) while excluding others (e.g. parental physical abuse and emotional abuse, peer bullying, and community violence) that were included in the present study.

The second goal of this study was to examine the proportion of adolescents who experienced different ACEs and were assigned to each HRB class. First, different types of ACEs were positively associated with different HRB patterns, and as the number of ACEs increased, the correlation effect increased. Toxic stress theory and cumulative risk models provide a useful framework for interpreting and understanding our findings. Toxic stress reactions occur when children experience intense, frequent, or prolonged adversity without adequate adult support, such as physical or emotional abuse, chronic neglect, and exposure to violence; the more adverse experiences in childhood, the greater the likelihood of later health problems, including substance abuse and depression [21]. For adverse events, the support for these theoretical arguments is relatively strong. For example, a previous study noted that young people who have experienced some type of childhood trauma, such as sexual, physical, or emotional abuse, tend to have a higher risk of alcohol and drug use disorders than those who have not [47]. Another study found that there was a strong association between exposure to childhood adversity and internalized and externalized behaviors in children after adjusting for sociodemographic factors and family income; only at higher levels of ACEs (three or more) was exposure to these adversities more likely to cause a child to display internalized or externalized behaviors that require professional attention compared to children with two or less [48].

### Gender differences

The final goal of this study was to examine the gender difference between ACEs and HRB patterns. In our analysis, females had noticeably higher odds of demonstrating “Self-harm” and “High all” than males at each ACE exposure level. Males, in contrast, were more likely to demonstrate “Unhealthy lifestyle” compared to females. Currently, there is a lack of research on gender differences in the association between ACEs and HRB patterns. However, some scholars have attempted to identify gender differences between ACEs and a single risky behavior [24, 25]. The overall results have not been uniform; for example, a national urban birth cohort study assessed that girls were more likely to develop externalizing behavioral problems after exposure to adversity, compared to boys [49]. Meanwhile, Pournaghash-Tehrani et al. [24] reported that the link between ACEs and suicidal ideation was stronger in girls than in boys, while another study found that suicidal ideation was more common in boys than girls exposed to emotional neglect [17]. In addition, a cross-sectional general health survey demonstrated that females exposed to ACE reported poorer mental health than males exposed to ACE, while males reported more substance use than females, and most outcomes did not differ significantly by sex [48].

### Strengths and limitations

This was a representative large-scale study linking ACEs and HRB patterns. One strength of this study was survey areas covering both urban and rural regions based on where our adolescent health research network was located, thus facilitating data collection. At the same time, due to the large sample size, we were able to conduct multivariable adjustment analysis, including gender differences. Then, we used a person-centered approach to identify distinct HRB patterns, which has the advantage of describing the heterogeneity of a population in terms of individual differences in a set of behaviors or characteristics, rather than only one variable. Additionally, all subtypes of ACEs were included in this study, with the exception of exposure to war/collective violence.

However, several limitations should be addressed. First, this study was conducted during the COVID-19 pandemic. A large body of literature has demonstrated that COVID-19 had a negative influence on adolescents’ behaviors [50–52]. We did not measure the impact of COVID-19 on participants, due to scope and data limitations. At that time, there were few COVID-19 cases in China and was under normalize epidemic management, the students in the area investigated by this study had resumed normal campus study and life, but the tension caused by pandemic may have influenced the results or caused recollection bias. Future research should minimize the impact of environmental factors, or may consider conducting the study post-pandemic. Second, as this was a cross-sectional study, we were unable to establish a causal relationship between variables. Currently, longitudinal studies have shown that the ACEs group has a higher percentage of adolescents in the high substance use category and much higher stability in this category [8]. At this position, there could also be bidirectional relations between ACEs and HRBs. For example, increased HRBs could also be related to more ACEs (e.g., substance use could lead to more problems in the parent–child relationship). Third, we relied on retrospective self-report measures by questionnaire. Therefore, this study may be affected by recall bias, common variance, and common method bias. Importantly, the results of a recent study found that recollections of childhood maltreatment and actual experiences of childhood maltreatment may have different outcomes for later health problems [53]. It was also recommended that future research should attempt to combine individuals’ recollections of previous ACEs with written case records. Finally, it may not be possible to generalize our findings to all Chinese adolescents, given our samples were focused on traditional school environment. However, a real-world study showed that some adolescents were absent from school and did not continue to study, which is important because the study showed that ACEs and risk behaviors are more common among individuals with lower educational achievement and socioeconomic status [17]. Future studies should be extended to community samples of adolescents.

## Conclusions and implications

Our results highlighted that different latent patterns of HRBs were related to ACEs. Females were more likely to have severe patterns than males. The current study, while not focused on clinical samples, can provide support for improving clinical care. Individuals with ACEs are the majority of clients served in public mental health and substance use treatment systems [8]. While many providers may conduct comprehensive interviews, including an in-depth assessment of past ACEs, for the most part, ACEs is not a major priority. Our results, although preliminary, also noted that any form of ACEs can trigger negative problems. Therefore, it also prompts us to consider the importance of trauma-informed therapy in clinical treatment and services. Furthermore, future work may explore protective and malleable factors based on individual (gender), family (economic level), peer education (school environment), etc., that contribute to mitigating the negative trajectory of ACEs, including reducing the clustering of multiple risk behaviors in the implementation of prevention and intervention strategies.

## Supplementary Information

**Additional file 1: Table S1.** Indicators of fit for models with one through six latent classes. **Table S2.** Average latent class probabilities for most likely latent class membership (row) by latent class (column). **Table S3.** The prevalence of specific HRBs within each class, n (%). **Table S4.** In the total sample, the relationship between numbers of ACEs and HRB patterns, Models 1 and 2. **Figure S1.** In the total sample, the relationship between the types of ACEs and different latent class of HRBs, Model 1: crude OR (95% CI). **Figure S2.** In the total sample, the relationship between the types of ACEs and different latent class of HRBs, Model 2: adjusted for grade, gender, residency, single child status, parents’ education level, family economic level, number of friends and self-evaluation of academic performance. **Figure S3.** In female. the association of ACEs with different latent class of HRBs, Model 3: adjusted for grade, gender, residency, single child status, parents’ education level, family economic level, number of friends, self-evaluation of academic performance and psychological symptoms. **Figure S4.** In male. the association of ACEs with different latent class of HRBs, Model 3: adjusted for grade, gender, residency, single child status, parents’ education level, family economic level, number of friends, self-evaluation of academic performance and psychological symptoms. **Figure S5.** In female. the relationship between numbers of ACEs and HRB patterns, Model 3. **Figure S6.** In male. the relationship between numbers of ACEs and HRB patterns, Model 3.

## Data Availability

The datasets analyzed in this study are not yet publicly available. Requests to access the datasets should be directed to 2004500039@ahmu.edu.cn.
