# Supplementary material for: Exploring associations of adverse childhood experiences with patterns of 11 health risk behaviors in Chinese adolescents: focus on gender differences
Source: Child Adolesc Psychiatry Ment Health. 2023 Feb 20;17:26. doi: 10.1186/s13034-023-00575-1 (PMC9940075; doi:10.1186/s13034-023-00575-1)
Supplement: Supplementary file 1 — Additional file 1: Table S1. Indicators of fit for models with one through six latent classes. Table S2. Average latent class probabilities for most likely latent class membership (row) by latent class (column). Table S3. The prevalence of specific HRBs within each class, n (%). Table S4. In the total sample, the relationship between numbers of ACEs and HRB patterns, Models 1 and 2. Figure S1. In the total sample, the relationship between the types of ACEs and different latent class of HRBs, Model 1: crude OR (95% CI). Figure S2. In the total sample, the relationship between the types of ACEs and different latent class of HRBs, Model 2: adjusted for grade, gender, residency, single child status, parents’ education level, family economic level, number of friends and self-evaluation of academic performance. Figure S3. In female. the association of ACEs with different latent class of HRBs, Model 3: adjusted for grade, gender, residency, single child status, parents’ education level, family economic level, number of friends, self-evaluation of academic performance and psychological symptoms. Figure S4. In male. the association of ACEs with different latent class of HRBs, Model 3: adjusted for grade, gender, residency, single child status, parents’ education level, family economic level, number of friends, self-evaluation of academic performance and psychological symptoms. Figure S5. In female. the relationship between numbers of ACEs and HRB patterns, Model 3. Figure S6. In male. the relationship between numbers of ACEs and HRB patterns, Model 3. [file 13034_2023_575_MOESM1_ESM.docx]

**Additional file 1**

*Table S1* Indicators of fit for models with one through six latent classes

| Model | *df* | *AIC* | *BIC* | *aBIC* | *Entropy* | *LMR-LRT* | *BLRT* | Class Probability |
| --- | --- | --- | --- | --- | --- | --- | --- | --- |
| 1 | 11 | 164854.54 | 164939.59 | 164904.64 | - | - | - | - |
| 2 | 23 | 149615.54 | 149793.38 | 149720.29 | 0.841 | <0.001 | <0.001 | 23.41/76.59 |
| 3 | 35 | 145203.43 | 145474.06 | 145362.83 | 0.780 | <0.001 | <0.001 | 20.13/17.13/64.74 |
| **4** | **47** | **142845.49** | **143208.91** | **143059.55** | **0.787** | <0.001 | <0.001 | **18.42/5.00/58.35/18.23** |
| 5 | 59 | 141967.84 | 142424.04 | 142236.55 | 0.777 | <0.001 | <0.001 | 4.98/7.60/11.65/17.95/57.82 |
| 6 | 71 | 141616.34 | 142165.33 | 141939.69 | 0.797 | <0.001 | <0.001 | 7.61/9.10/15.72/2.53/12.07/52.96 |
| d*f*, degrees of freedom; AIC, Akaike Information Criteria; BIC, Bayesian Information Criteria; aBIC, Adjusted Bayesian Information Criteria; LMR-LRT, Lo-Mendell-Rubin Likelihood Ratio; BLRT, Bootstrapped Likelihood Ratio Tests. | | | | | | | | |

*Table S2* Average latent class probabilities for most likely latent class membership (row) by latent class (column).

|  | Class1 | Class2 | Class3 | Class4 |
| --- | --- | --- | --- | --- |
| Class1: Low all | 0.919 | 0.058 | 0.024 | 0.000 |
| Class2: Unhealthy lifestyle | 0.155 | 0.803 | 0.030 | 0.012 |
| Class3: Self-harm | 0.056 | 0.034 | 0.868 | 0.042 |
| Class4: High all | 0.000 | 0.016 | 0.103 | 0.881 |

*Table S3* The prevalence of specific HRBs within each class, n(%).

| HRBs | Class1  (n_1_=9833) | Class2  (n_2_=3073) | Class3  (n_3_=3104) | Class4  (n_4_=843) |
| --- | --- | --- | --- | --- |
| Smoking | 63(0.6) | 187(6.1) | 104(3.4) | 251(29.8) |
| Alcohol use | 286(2.9) | 629(20.5) | 491(15.8) | 418(49.6) |
| Takeaway | 150(1.5) | 1309(42.6) | 127(4.1) | 585(69.4) |
| Fast food | 472(4.8) | 1707(55.5) | 382(12.3) | 638(75.7) |
| Carbonated drink | 507(5.2) | 1993(64.9) | 480(15.5) | 611(72.5) |
| Sugared drink | 1728(17.6) | 2267(73.8) | 963(31.0) | 635(75.3) |
| Screen time | 900(9.2) | 891(29.0) | 432(13.9) | 443(52.6) |
| NSSI | 1159(11.8) | 740(24.1) | 2363(76.1) | 608(72.1) |
| Suicide ideation | 893(9.1) | 538(17.5) | 3042(98.0) | 805(95.5) |
| Suicide plan | 48(0.5) | 15(0.5) | 1887(60.8) | 790(93.7) |
| Suicide attempt | 14(0.1) | 20(0.7) | 698(22.5) | 528(62.6) |

*Table S4* In the total sample, the relationship between numbers of ACEs and HRB patterns, Models 1 and 2.

| Variables | Class2^*^ | | Class 3^*^ | | Class 4^*^ | |
| --- | --- | --- | --- | --- | --- | --- |
|  | *OR* (95% *CI*) | p-value | *OR* (95% *CI*) | p-value | *OR* (95% *CI*) | p-value |
| Model 1 |  |  |  |  |  |  |
| 5-8 | 1.67(1.44-1.92) | <0.001 | 13.14(11.08-15.59) | <0.001 | 7.65(6.06-9.64) | <0.001 |
| 3-4 | 1.30(1.15-1.47) | <0.001 | 5.27(4.47-6.23) | <0.001 | 1.95(1.52-2.49) | <0.001 |
| 1-2 | 1.15(1.03-1.28) | 0.015 | 2.17(1.84-2.57) | <0.001 | 0.95(0.74-1.21) | 0.665 |
| 0 | 1.00 |  | 1.00 |  | 1.00 |  |
| Model 2 |  |  |  |  |  |  |
| 5-8 | 1.86(1.61-2.16) | <0.001 | 12.47(10.49-14.83) | <0.001 | 8.30(6.54-10.54) | <0.001 |
| 3-4 | 1.43(1.26-1.62) | <0.001 | 5.19(4.38-6.14) | <0.001 | 2.23(2.74-2.87) | <0.001 |
| 1-2 | 1.23(1.10-1.38) | <0.001 | 2.21(1.86-2.61) | <0.001 | 1.09(0.85-1.40) | 0.516 |
| 0 | 1.00 |  | 1.00 |  | 1.00 |  |
| Class1: Low all, Class2: Unhealthy lifestyle, Class3: Self-harm, Class4: High all. ^*^ Class 1 was used as the reference category; Model 1: Crude OR (95% CI). Model 2: adjusted for grade, gender, residency, single child status, parents’ education level, family economic level, number of friends and self-evaluation of academic performance. | | | | | | |


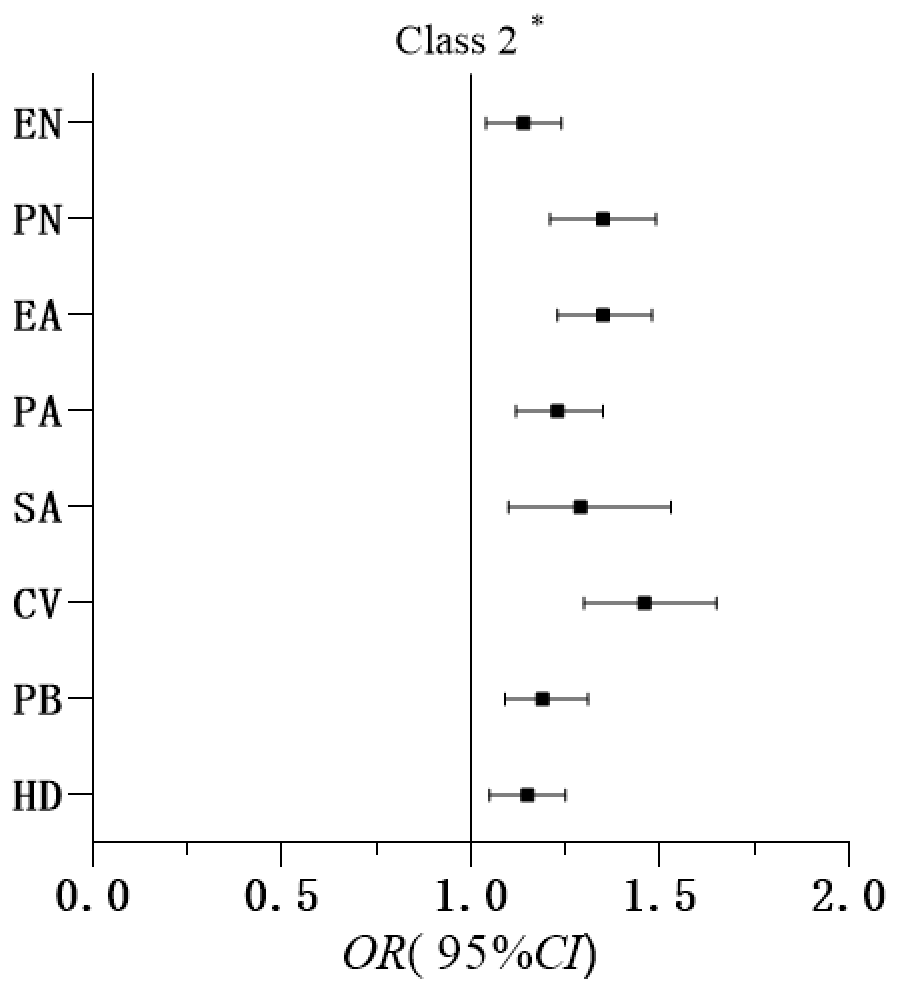

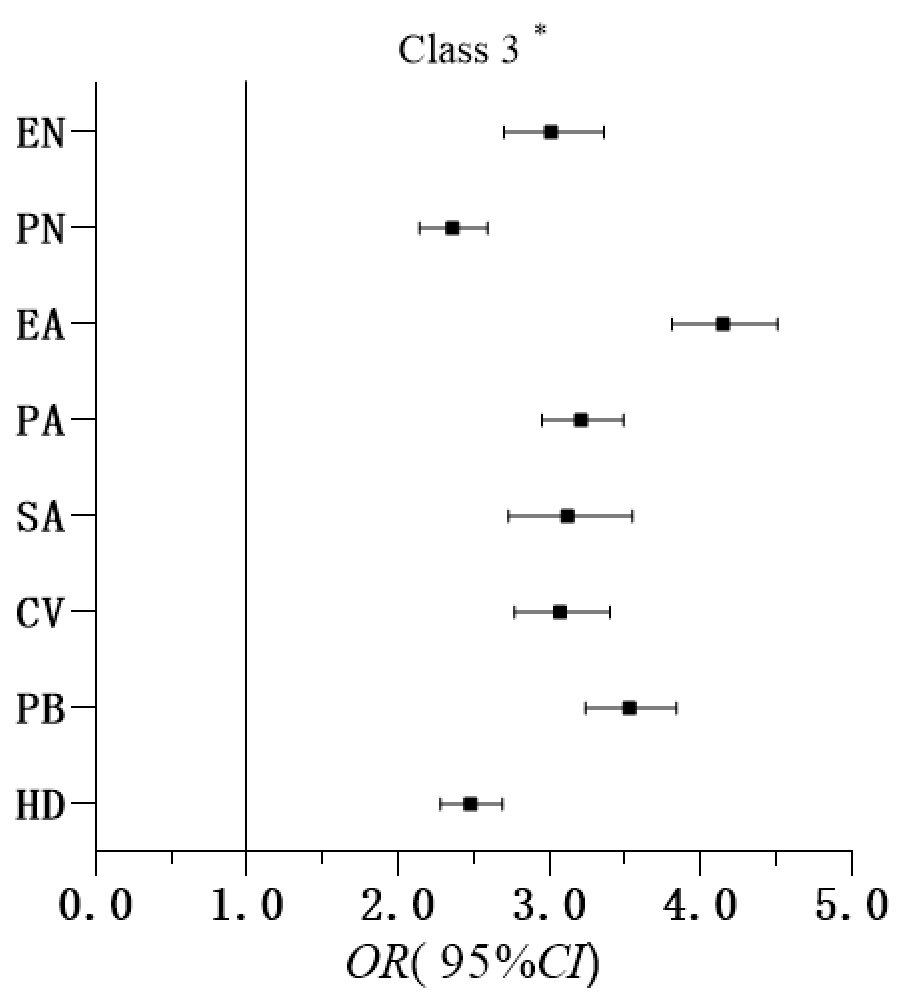

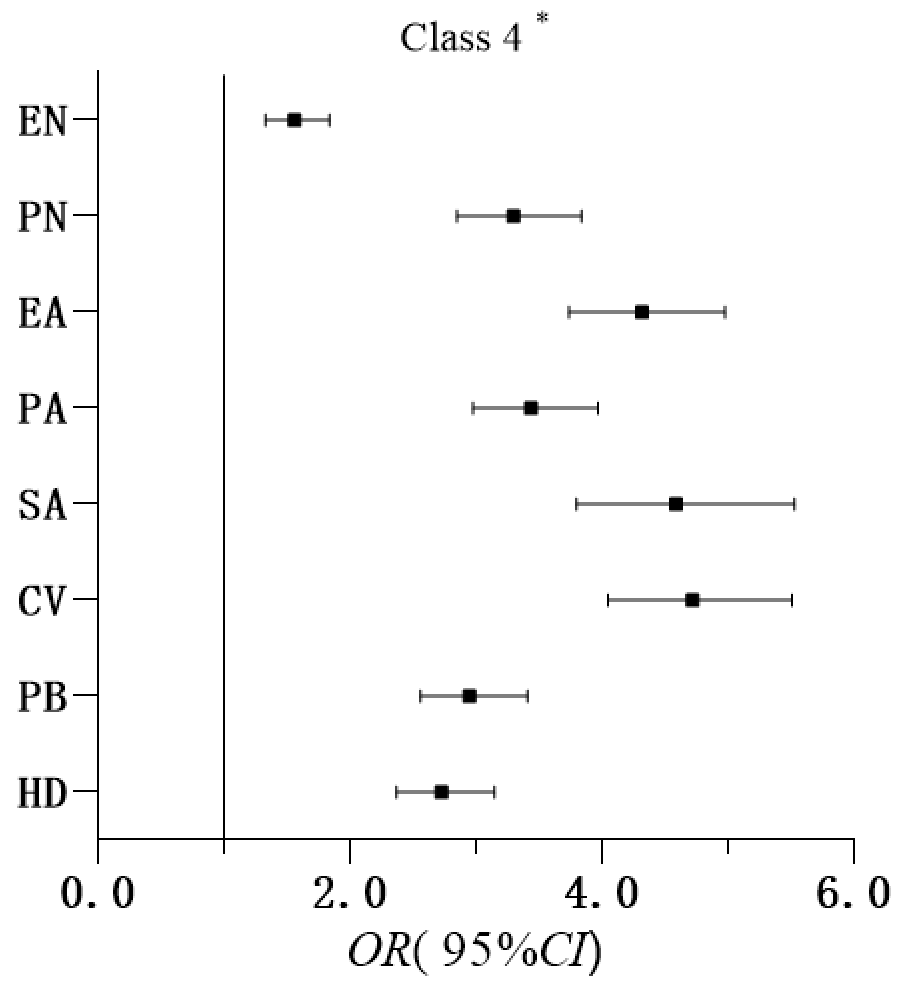


*Figure S1* In the total sample, the relationship between the types of ACEs and different latent class of HRBs, Model 1: crude *OR* (95% *CI*). Class1: Low all, Class2: Unhealthy lifestyle, Class3: Self-harm, Class4: High all. ***** Class 1 was used as the reference category; EN=Emotional neglect, PN=Physical neglect, EA=Emotional abuse, PA=Physical abuse, SA=Sexual abuse, CV=Community violence, PB=Peer bullying, HD=Household dysfunction.


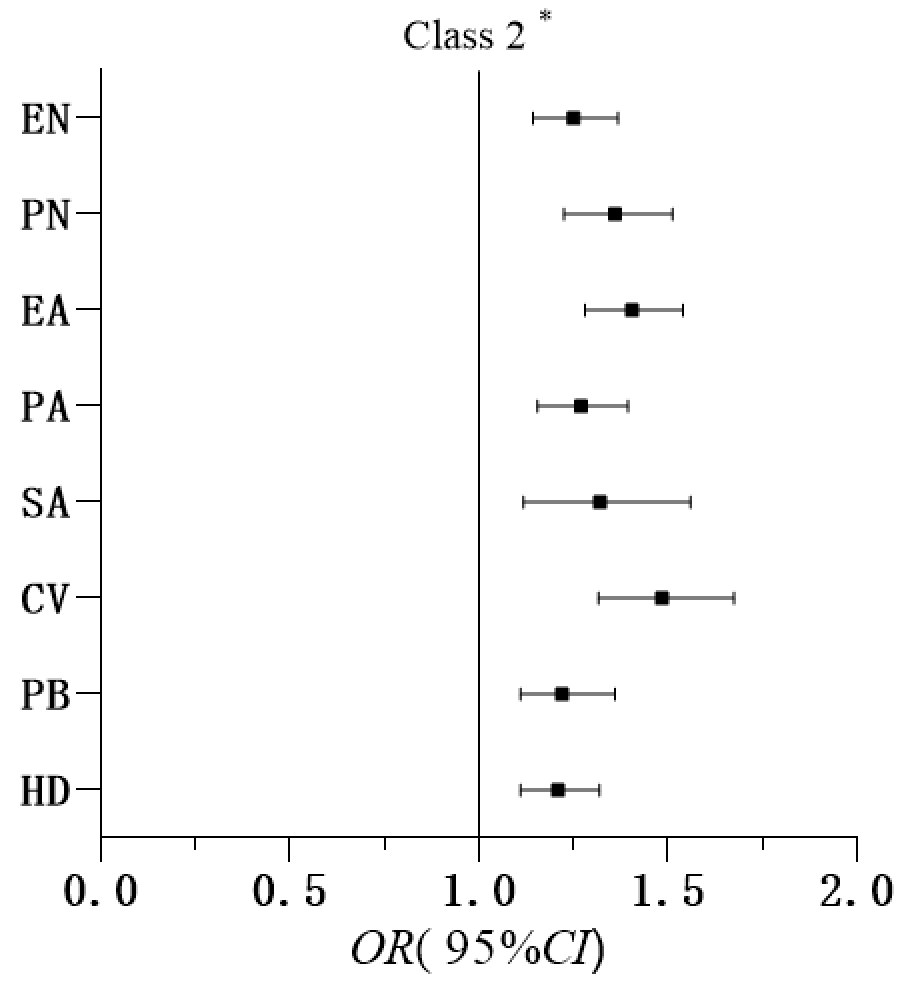

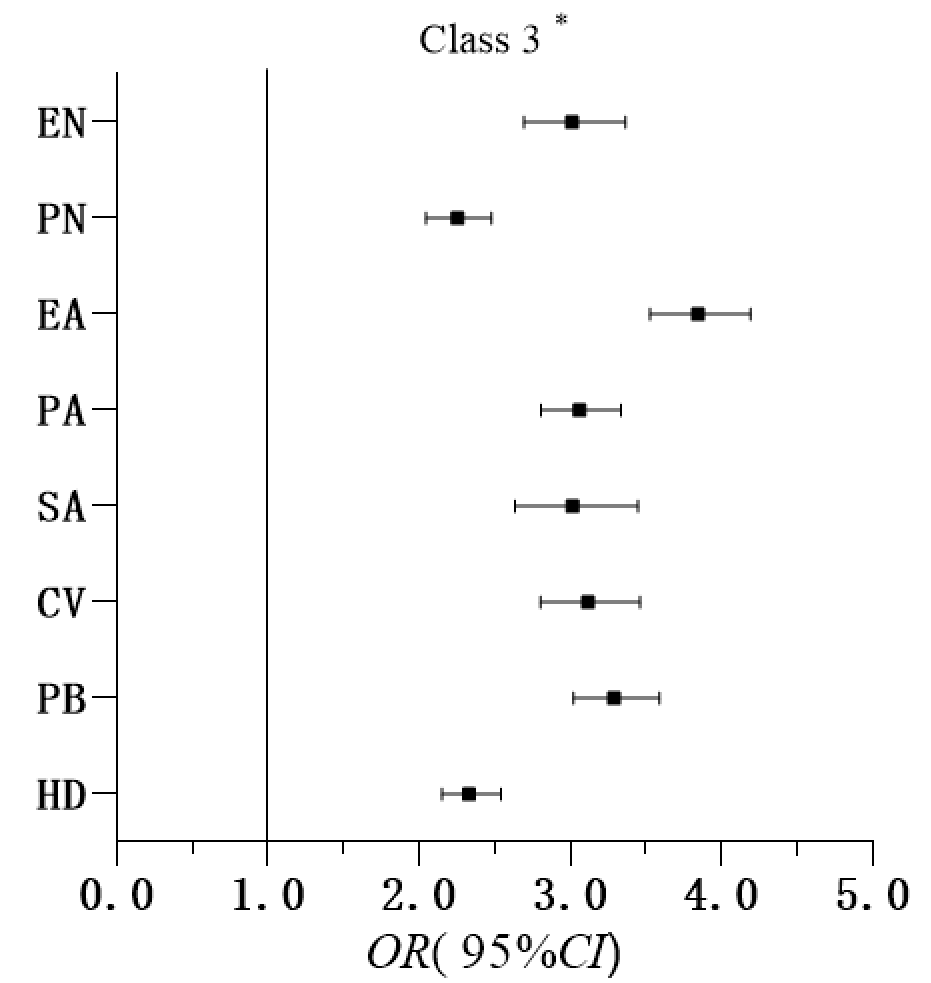

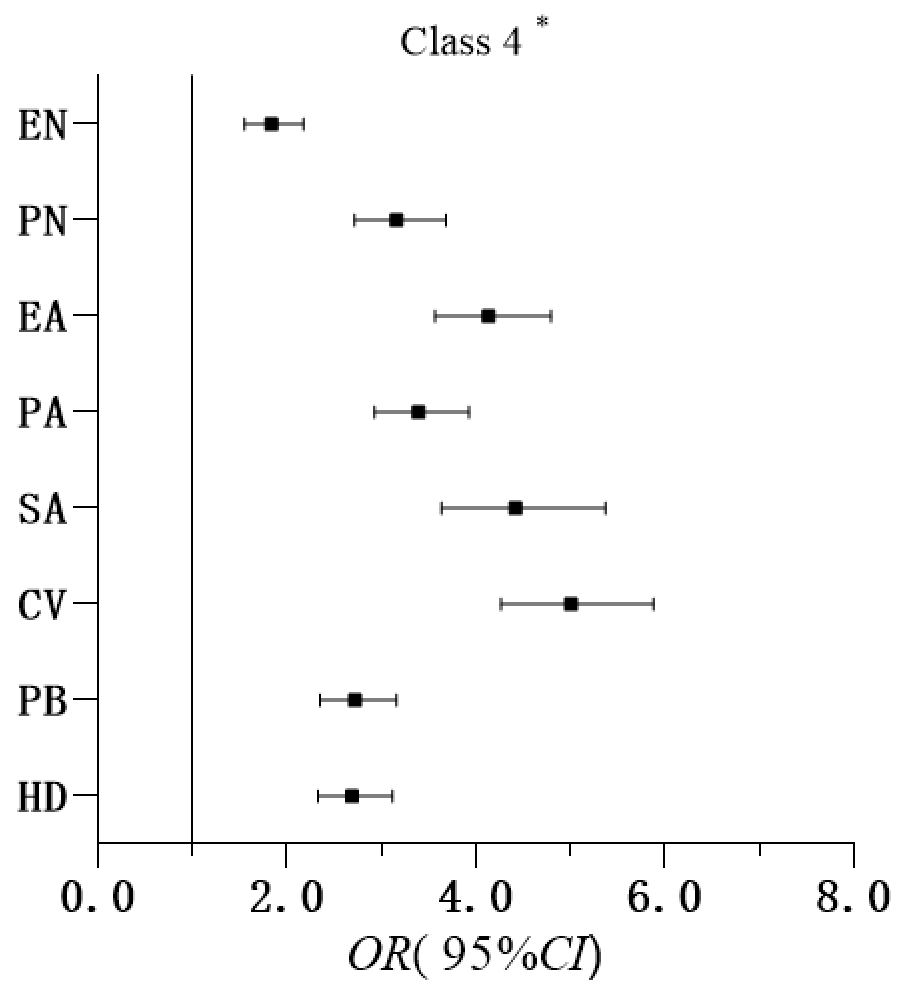


*Figure S2* In the total sample, the relationship between the types of ACEs and different latent class of HRBs, Model 2: adjusted for grade, gender, residency, single child status, parents’ education level, family economic level, number of friends and self-evaluation of academic performance. Class1: Low all, Class2: Unhealthy lifestyle, Class3: Self-harm, Class4: High all. **^*^** Class 1 was used as the reference category; EN=Emotional neglect, PN=Physical neglect, EA=Emotional abuse, PA=Physical abuse, SA=Sexual abuse, CV=Community violence, PB=Peer bullying, HD=Household dysfunction.


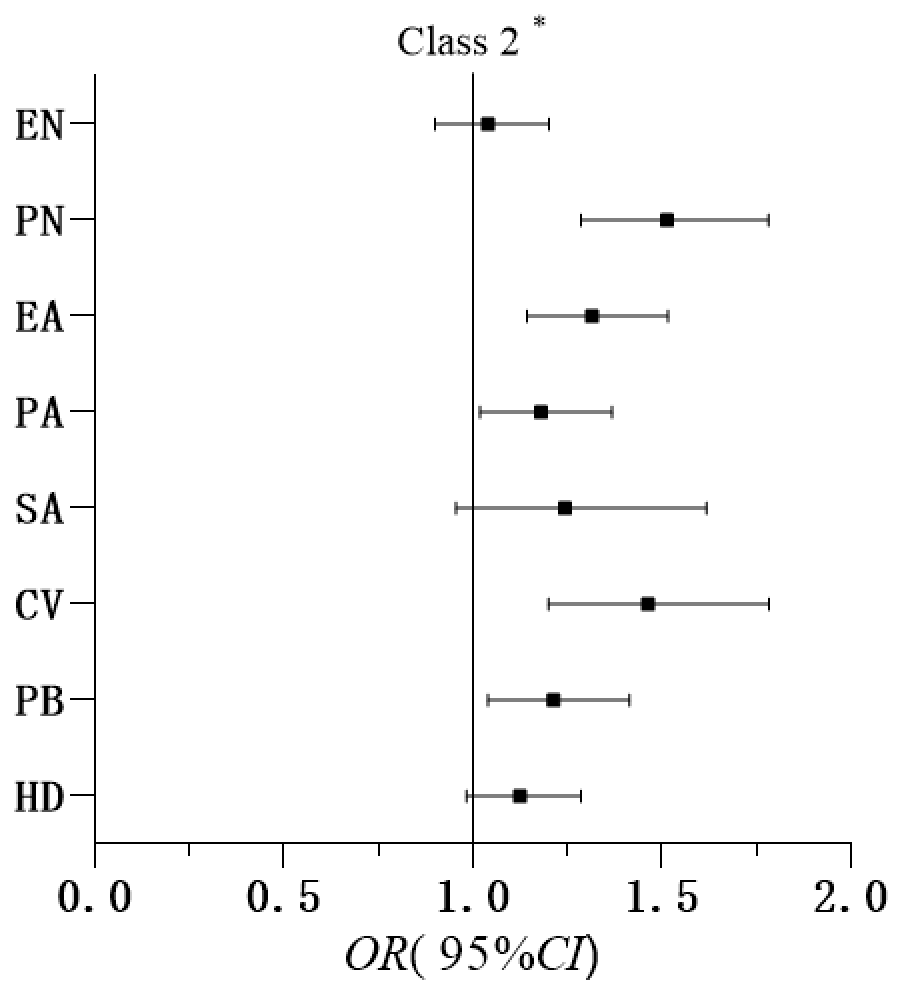

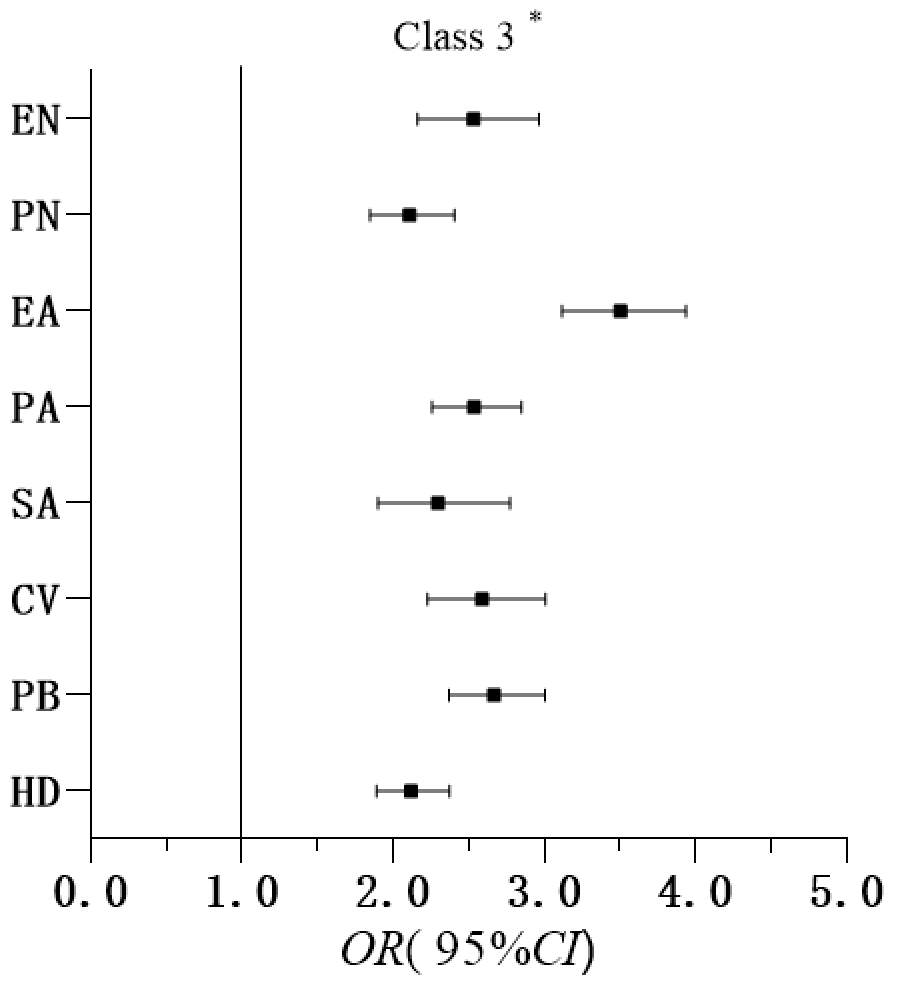

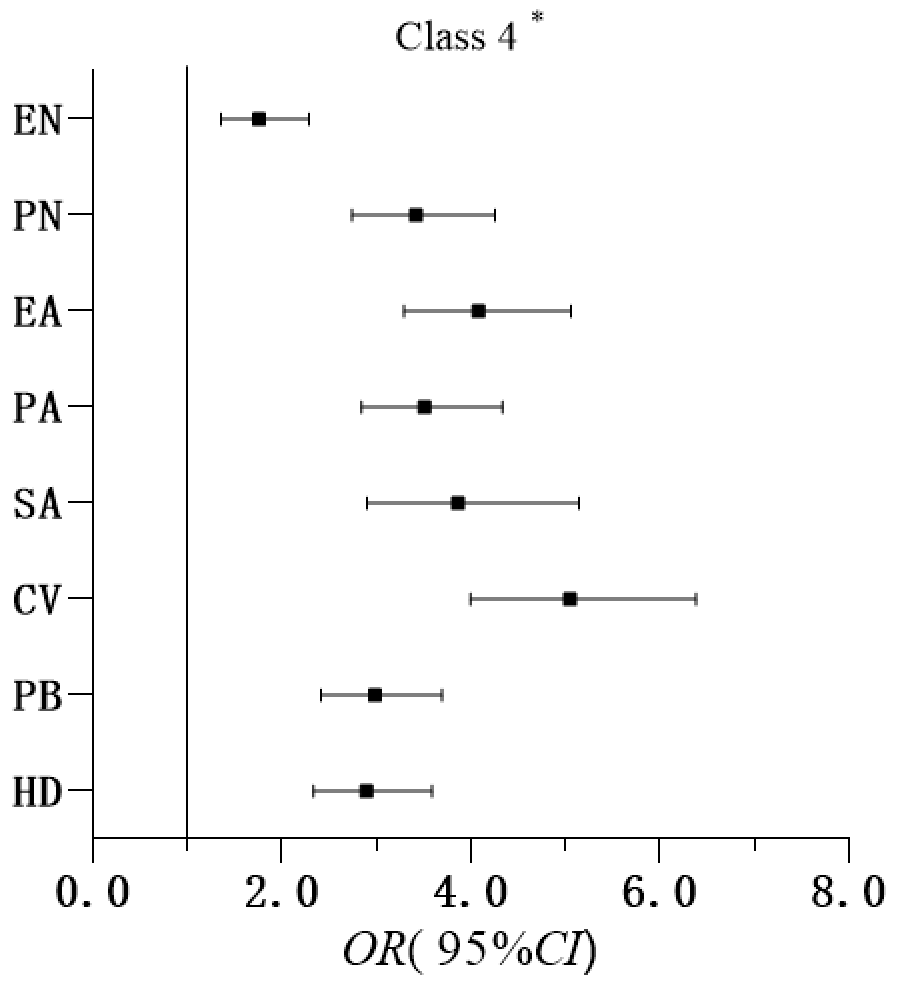


*Figure S3* In female. the association of ACEs with different latent class of HRBs, Model 3: adjusted for grade, gender, residency, single child status, parents’ education level, family economic level, number of friends, self-evaluation of academic performance and psychological symptoms. Class1: Low all, Class2: Unhealthy lifestyle, Class3: Self-harm, Class4: High all. ***** Class 1 was used as the reference category; EN=Emotional neglect, PN=Physical neglect, EA=Emotional abuse, PA=Physical abuse, SA=Sexual abuse, CV=Community violence, PB=Peer bullying, HD=Household dysfunction.


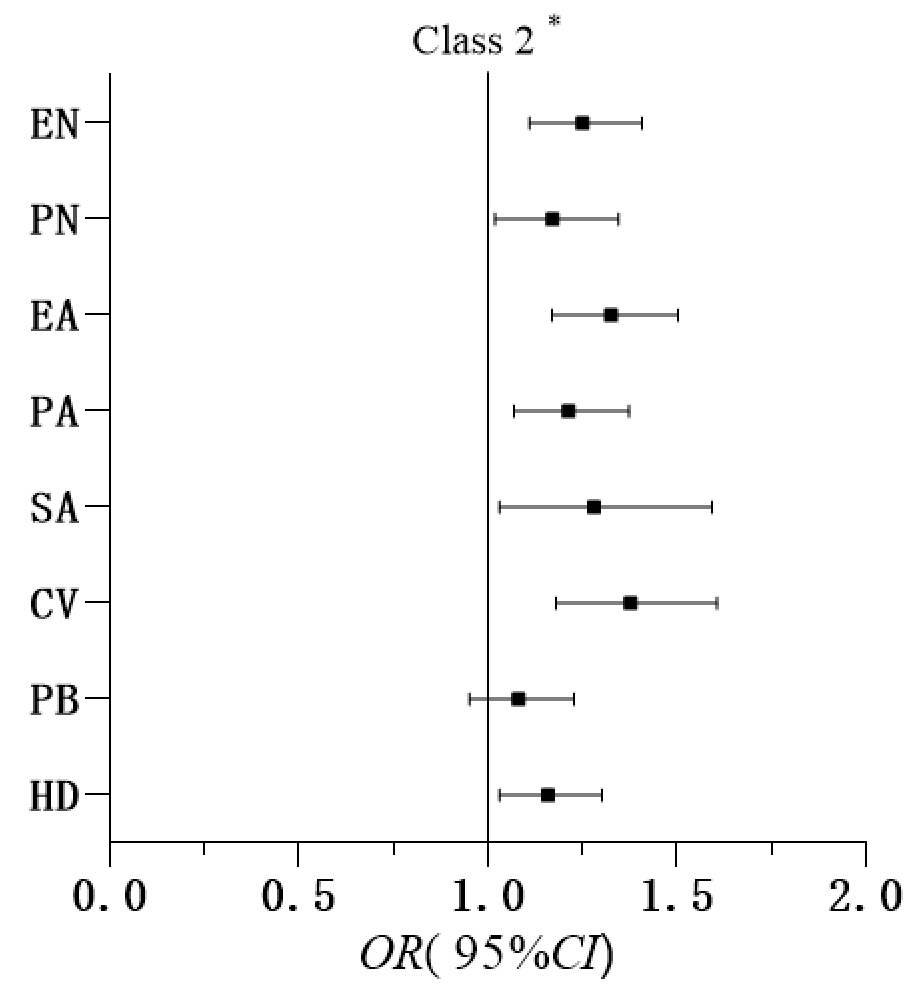

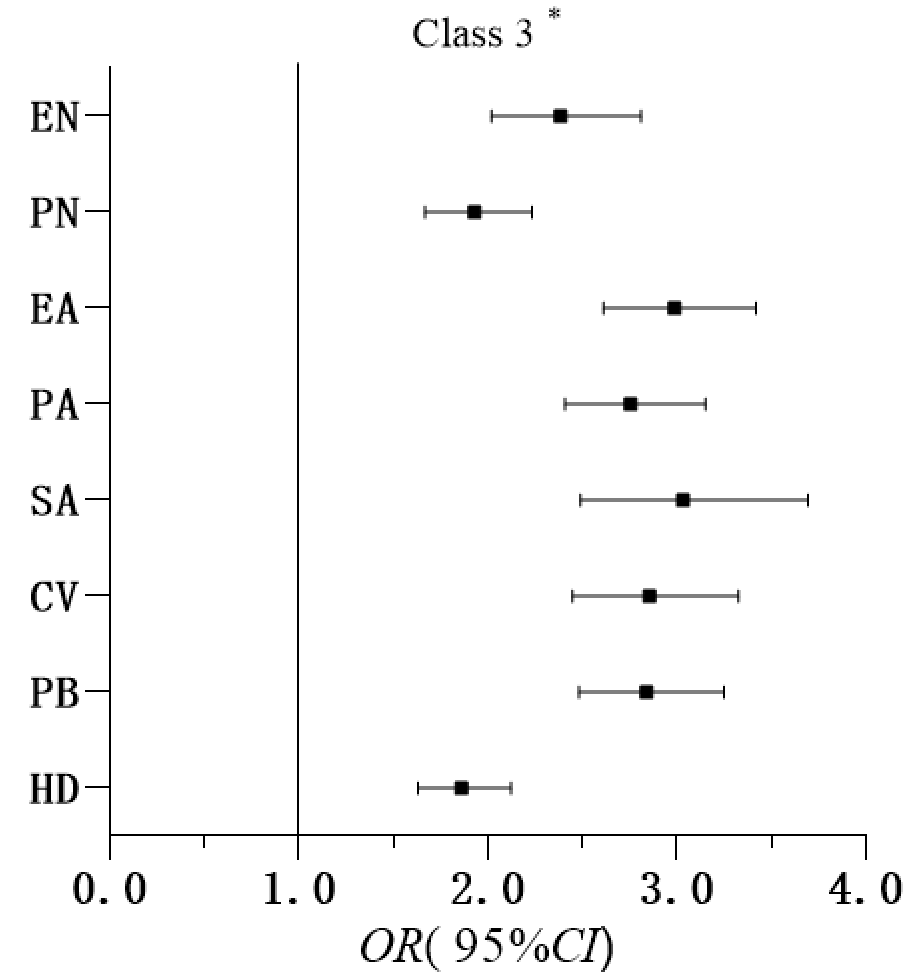

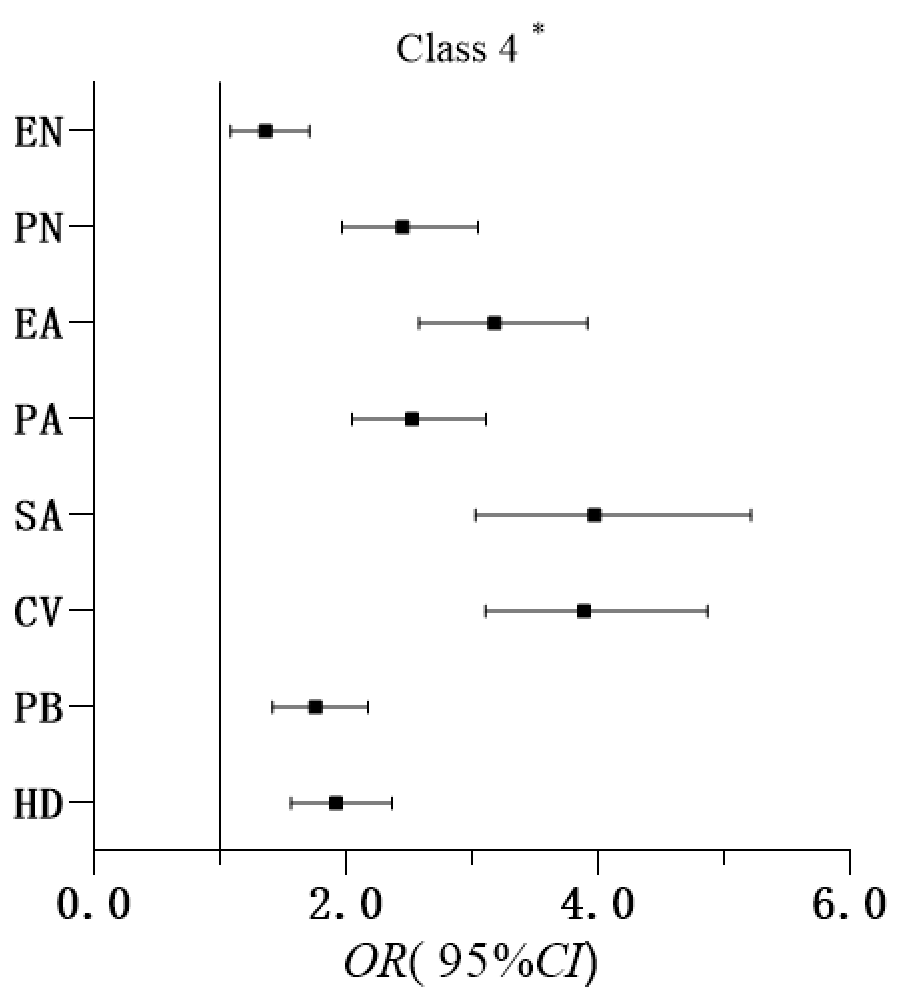


*Figure S4* In male. the association of ACEs with different latent class of HRBs, Model 3: adjusted for grade, gender, residency, single child status, parents’ education level, family economic level, number of friends, self-evaluation of academic performance and psychological symptoms. Class1: Low all, Class2: Unhealthy lifestyle, Class3: Self-harm, Class4: High all. * Class 1 was used as the reference category; EN=Emotional neglect, PN=Physical neglect, EA=Emotional abuse, PA=Physical abuse, SA=Sexual abuse, CV=Community violence, PB=Peer bullying, HD=Household dysfunction.


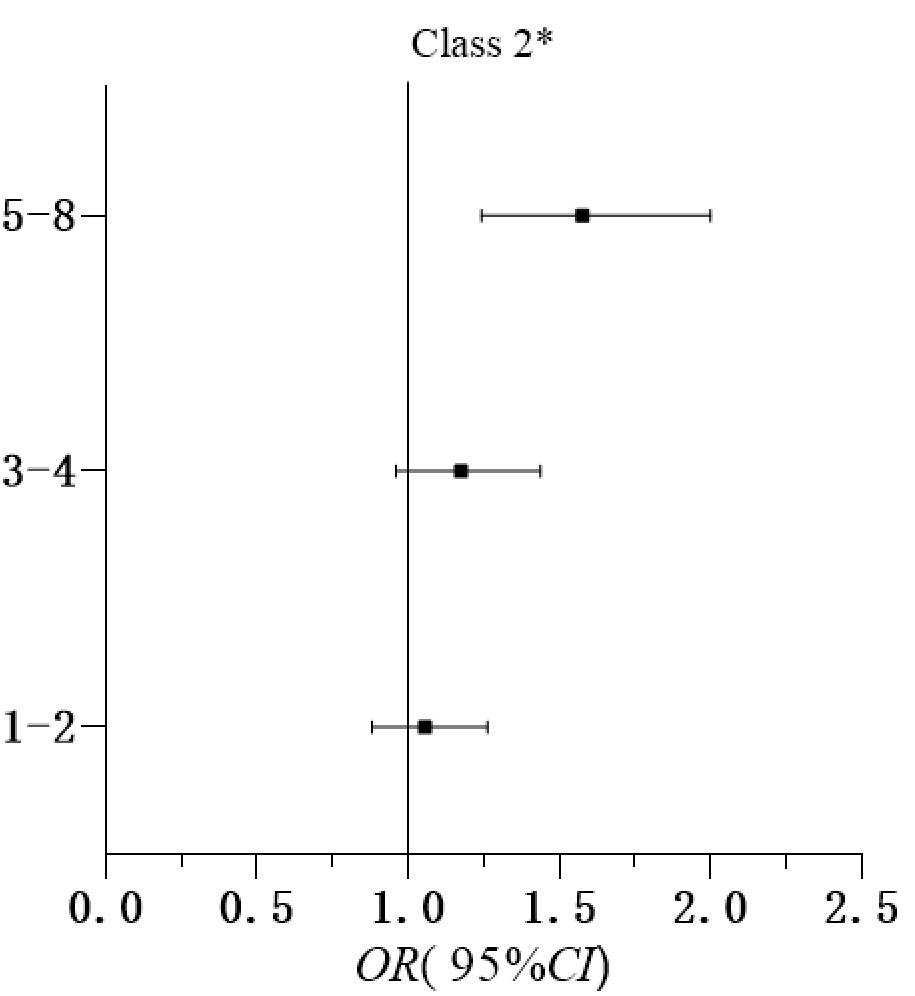

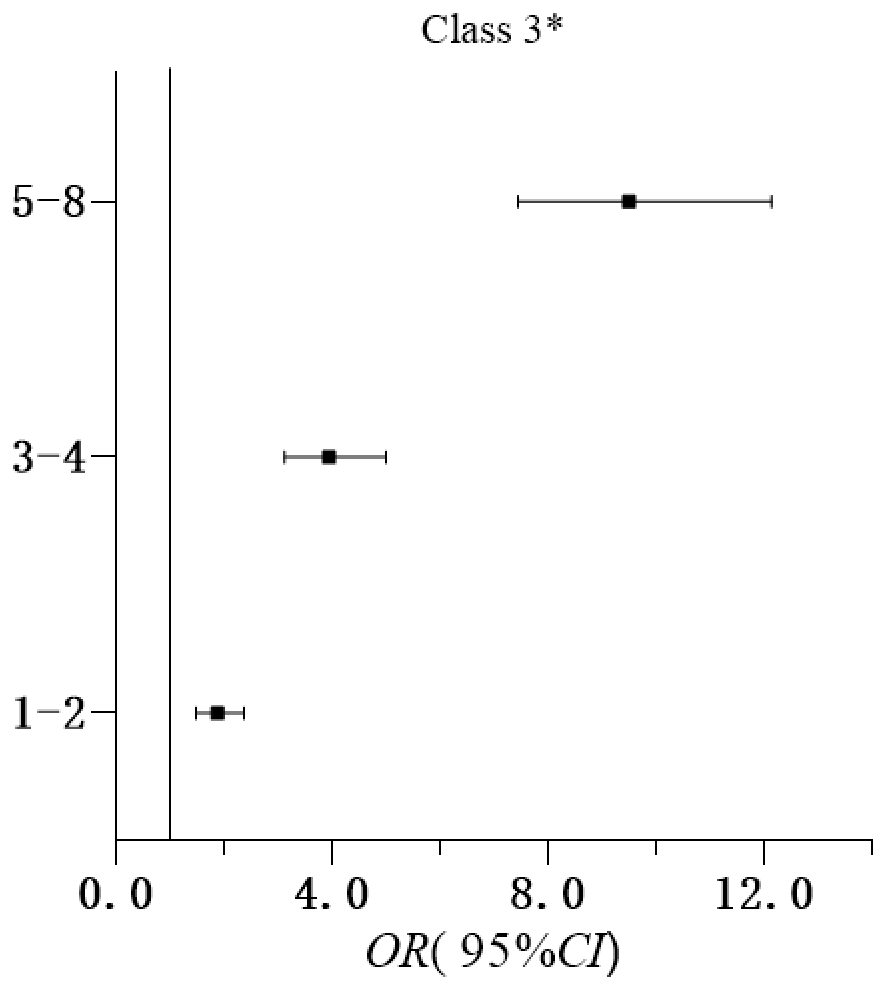

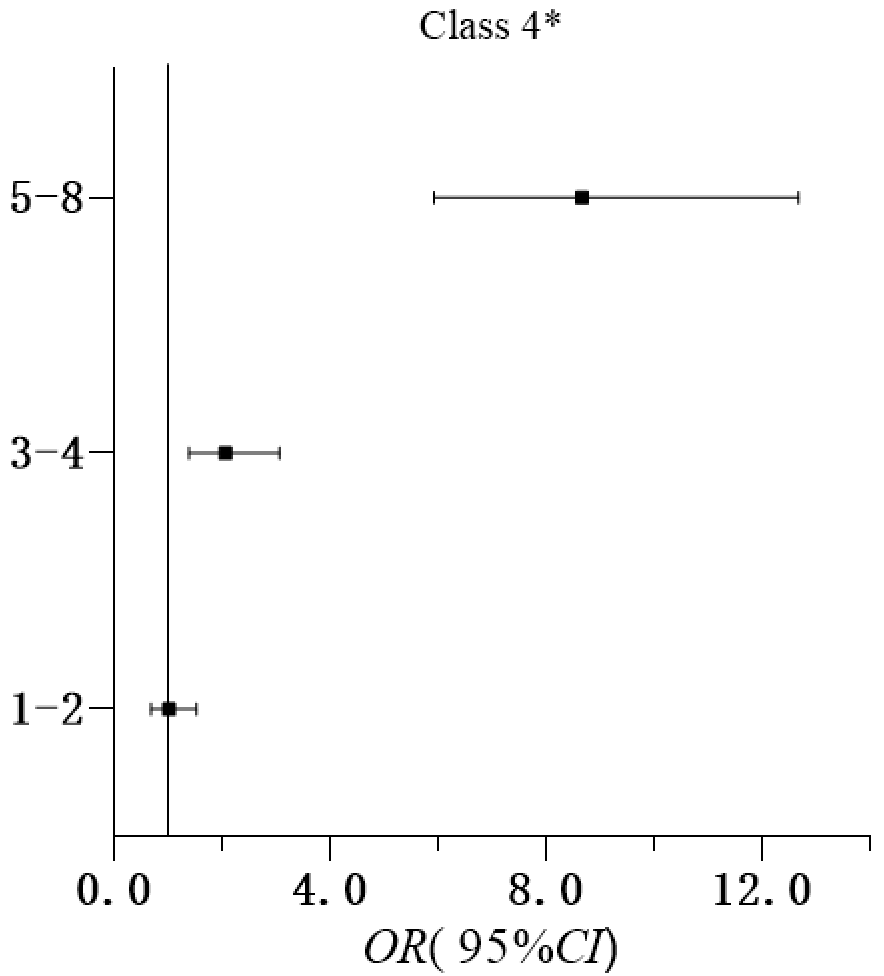


*Figure S5* In female. the relationship between numbers of ACEs and HRB patterns, Model 3. Adjusted for grade, gender, residency, single child status, parents’ education level, family economic level, number of friends, self-evaluation of academic performance and psychological symptoms. Class1: Low all, Class2: Unhealthy lifestyle, Class3: Self-harm, Class4: High all. ^*^ Class 1 was used as the reference category.


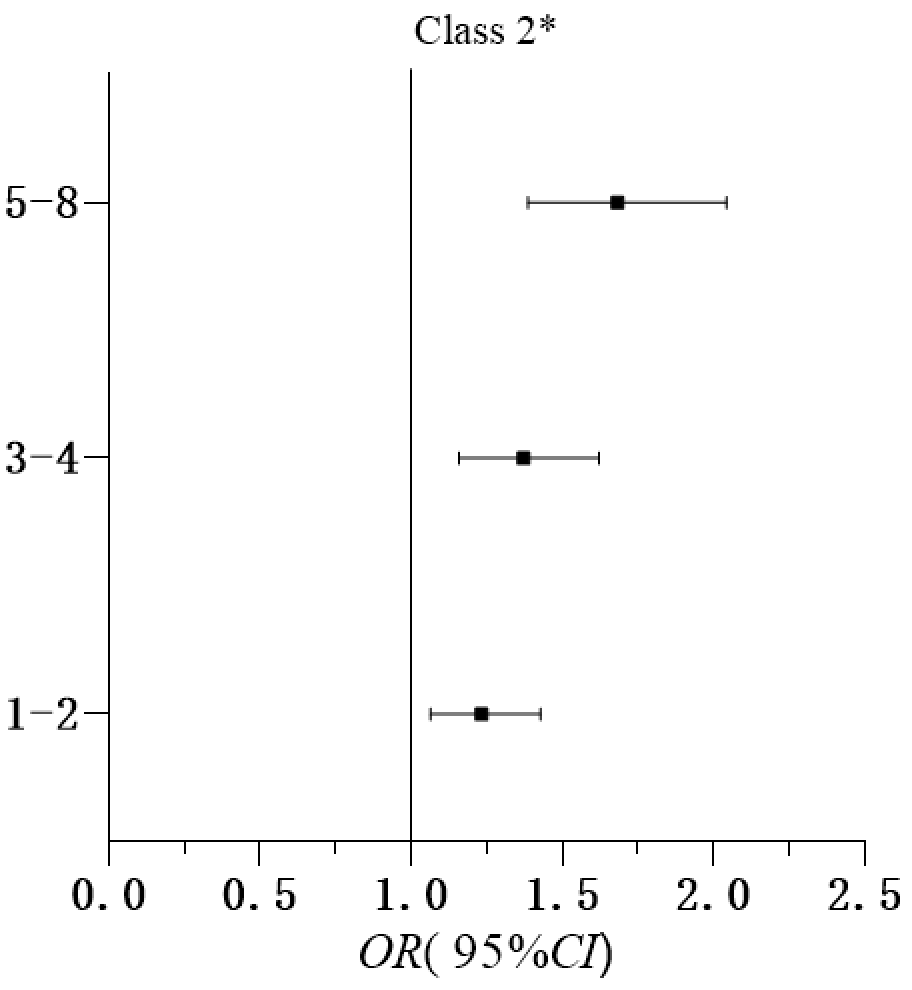

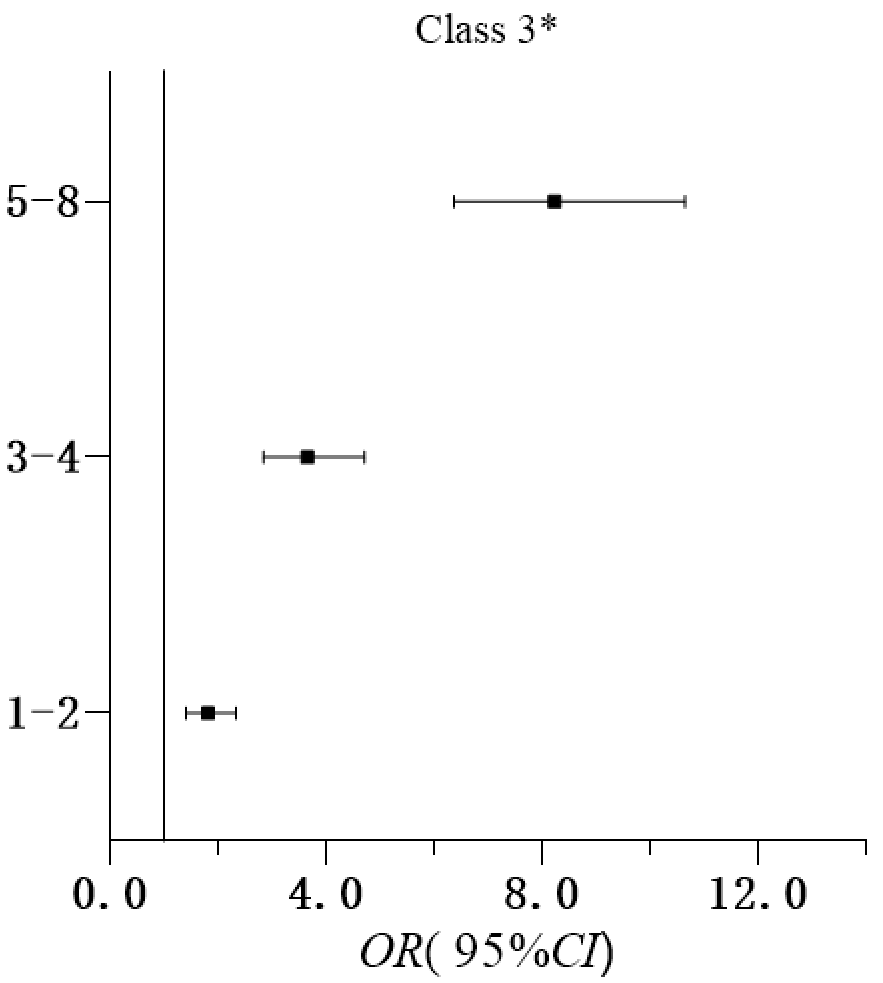

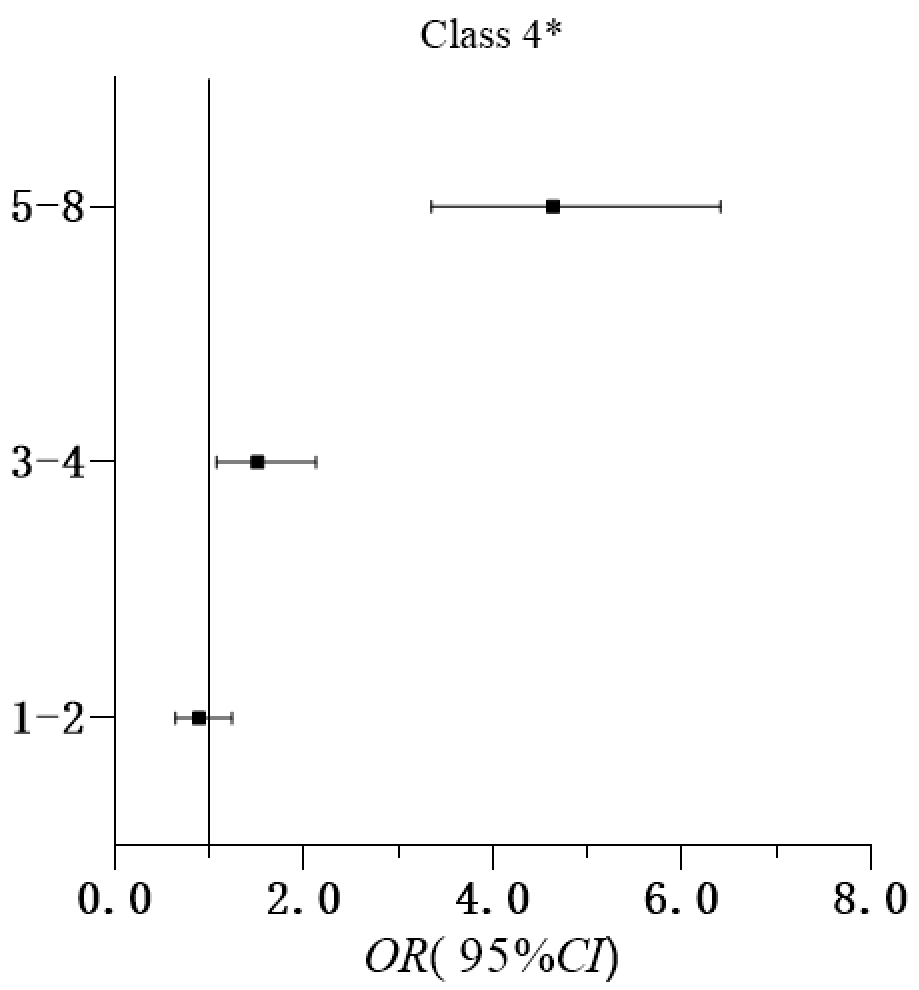


*Figure S6* In male. the relationship between numbers of ACEs and HRB patterns, Model 3. Adjusted for grade, gender, residency, single child status, parents’ education level, family economic level, number of friends, self-evaluation of academic performance and psychological symptoms. Class1: Low all, Class2: Unhealthy lifestyle, Class3: Self-harm, Class4: High all. ^*^ Class 1 was used as the reference category.
